# Multiple regulatory mechanisms of the biological function of NRF3 (NFE2L3) control cancer cell proliferation

**DOI:** 10.1038/s41598-017-12675-y

**Published:** 2017-10-02

**Authors:** A. M. Masudul Azad Chowdhury, Hiroki Katoh, Atsushi Hatanaka, Hiroko Iwanari, Nanami Nakamura, Takao Hamakubo, Tohru Natsume, Tsuyoshi Waku, Akira Kobayashi

**Affiliations:** 1 Laboratory for Genetic Code, Graduate School of Life and Medical Sciences, Doshisha University, Kyotanabe Kyoto, Japan; 2 Department of Quantitative Biology and Medicine, Research Center for Advanced Science and Technology, The University of Tokyo, Tokyo, Japan; 3 National Institutes of Advanced Industrial Science and Technology, Biological Information Research Center (JBIRC), Tokyo, Japan

## Abstract

Accumulated evidence suggests a physiological relationship between the transcription factor NRF3 (NFE2L3) and cancers. Under physiological conditions, NRF3 is repressed by its endoplasmic reticulum (ER) sequestration. In response to unidentified signals, NRF3 enters the nucleus and modulates gene expression. However, molecular mechanisms underlying the nuclear translocation of NRF3 and its target gene in cancer cells remain poorly understood. We herein report that multiple regulation of NRF3 activities controls cell proliferation. Our analyses reveal that under physiological conditions, NRF3 is rapidly degraded by the ER-associated degradation (ERAD) ubiquitin ligase HRD1 and valosin-containing protein (VCP) in the cytoplasm. Furthermore, NRF3 is also degraded by β-TRCP, an adaptor for the Skp1-Cul1-F-box protein (SCF) ubiquitin ligase in the nucleus. The nuclear translocation of NRF3 from the ER requires the aspartic protease DNA-damage inducible 1 homolog 2 (DDI2) but does not require inhibition of its HRD1-VCP-mediated degradation. Finally, NRF3 mediates gene expression of the cell cycle regulator U2AF homology motif kinase 1 (UHMK1) for cell proliferation. Collectively, our study provides us many insights into the molecular regulation and biological function of NRF3 in cancer cells.

## Introduction

The transcription factor NRF3 (NF-E2-related factor 3 or NFE2L3) belongs to the cap ‘n’ collar (CNC) family comprising NRF1 and NRF2^1–4^. The physiological roles of NRF3 were unknown, in part because *Nrf3* knockout mice do not show apparent abnormalities^5–8^. Recently, a physiological relationship between NRF3 and cancers has been reported. The human cancer genome project has identified *NRF3* as one of the 127 significantly mutated genes^9^ and reports its significant gene induction in human cancers including colorectal adenocarcinoma^10–12^. Extensive biochemical studies have elucidated a part of the regulatory mechanisms of NRF3. Under physiological conditions, the transcriptional activity of NRF3 is repressed by its sequestration in the endoplasmic reticulum (ER), thereby preventing its unnecessary gene expression^13^. Upon exposure to a stress and/or a signal, which has not yet been identified, NRF3 translocates into the nucleus and exerts its transcriptional activity through the antioxidant response element (ARE) or Maf recognition elements (MARE) by heterodimerizing with small Maf proteins. These observations imply that NRF3 functions as an inducible transcription factor in response to certain activation signal(s). To understand the comprehensive biological function of NRF3 in cancer cells, further elucidation of its regulatory mechanisms, including its nuclear entry from the ER, and the identification of its target gene(s) are indispensable.

The ubiquitin proteasome system (UPS) mediates the turnover of proteins in a variety of biological processes such as cell cycle progression, signal transduction and transcription^14^. The proteasome degrades substrate proteins that are conjugated with the polyubiquitin chain degradation signal by way of the E3 ubiquitin ligase. The key feature of ubiquitin-mediated degradation is that it is rapid and specific. This allows cells to mediate their regulatory pathways in response to intrinsic and extrinsic signals.

The ER-associated protein degradation (ERAD) system removes misfolded or unassembled proteins for protein quality control in the ER. The molecular basis of ERAD degradation comprises three sequential steps: ubiquitination by specific ubiquitin ligases, substrate transportation from the ER to the cytoplasm (dislocation), and proteolysis by the proteasome^15^. HRD1 (also known as synoviolin), which is conserved between humans and yeast, is an ERAD ubiquitin ligase^16,17^. HRD1, with the adaptor SEL1L, conjugates a polyubiquitin chain to soluble, ER-luminal substrates and integral membrane proteins^18^. Consequently, the ubiquitinated proteins are recognized by p97/valosin-containing protein (VCP) and are transported to proteasome, resulting in their rapid degradation^18–20^.

The β-transducin repeat-containing protein (β-TRCP) is one of the F-box proteins of the SKP1-Cullin 1-F-box protein (SCF) E3 ligase complexes^21^. F-box proteins, in complex with the scaffold protein Cullin1 (Cul1) and S phase kinase associated protein 1 (SKP1), function as an adaptor to determine substrate specificity. β-TRCP regulates numerous cellular processes by mediating the stability of target proteins including cell cycle regulators, pro-apoptotic regulators and transcription factors. Mammals express two paralogs of β-TRCP, β-TRCP1 and β-TRCP2, which exhibit functional redundancy (thus, the paralogs will be referred to here as β-TRCP).

The U2AF Homology Motif Kinase 1 (UHMK1, also known as KIS1), which is a serine/threonine protein kinase, controls the cell cycle through the tumor suppressor p27Kip1 (cyclin-dependent kinase inhibitor)^22,23^. It phosphorylates p27Kip1 on Ser10, resulting in its cytoplasmic export and, ultimately, cell cycle progression. UHMK1 is activated by mitogens during G(0)/G(1), and the expression of UHMK1 overcomes growth arrest that is induced by p27Kip1. Alternatively, an siRNA-mediated *UHMK1* knockdown undergoes growth arrest by reducing p27Kip1 phosphorylation.

We herein describe multiple regulatory mechanisms of the biological function of NRF3. The turnover of NRF3 is regulated by two distinct proteasomal degradation mechanisms by HRD1-VCP and β-TRCP in the cytoplasm and the nucleus, respectively. The nuclear translocation of NRF3 from the ER sequestration requires the aspartic protease DDI2 but does not require the inhibition of HRD1-VCP-mediated NRF3 degradation in the cytoplasm. NRF3 promotes cancer cell proliferation by inducing the gene expression of the cell cycle regulator UHMK1. Altogether, our findings uncover that NRF3 under these multiple regulations causes the proliferation of colon cancer cells.

## Results

### HRD1 and VCP regulate the cytoplasmic degradation of NRF3

To elucidate the mechanisms underlying the molecular regulation of NRF3, we first conducted proteome analysis to identify the NRF3-associated proteins, as described previously^24^. The NRF3 complexes were immunopurified from the cell extract of HEK293 cells that were transiently expressing NRF3-Flag by using an anti-flag antibody. The resultant NRF3 complexes were subjected to liquid chromatography-tandem mass spectrometry (LC-MS/MS). Consequently, we succeeded in identifying several factors including proteasome subunits and a transcriptional mediator (Supplementary Table 1). Among these factors, we focused on the ubiquitin ligase-related factors VCP and SKP1 because we had previously discovered that the NRF3-related factor NRF1 is degraded by two distinct E3 ubiquitin ligase complexes (VCP-HRD1 and SKP1-β-TRCP) in the cytoplasm and nucleus, respectively^24^. This observation allowed us to form the hypothesis that the stability of NRF3 is also regulated by these E3 ubiquitin ligases, similar to that of NRF1.

**Figure 1:**
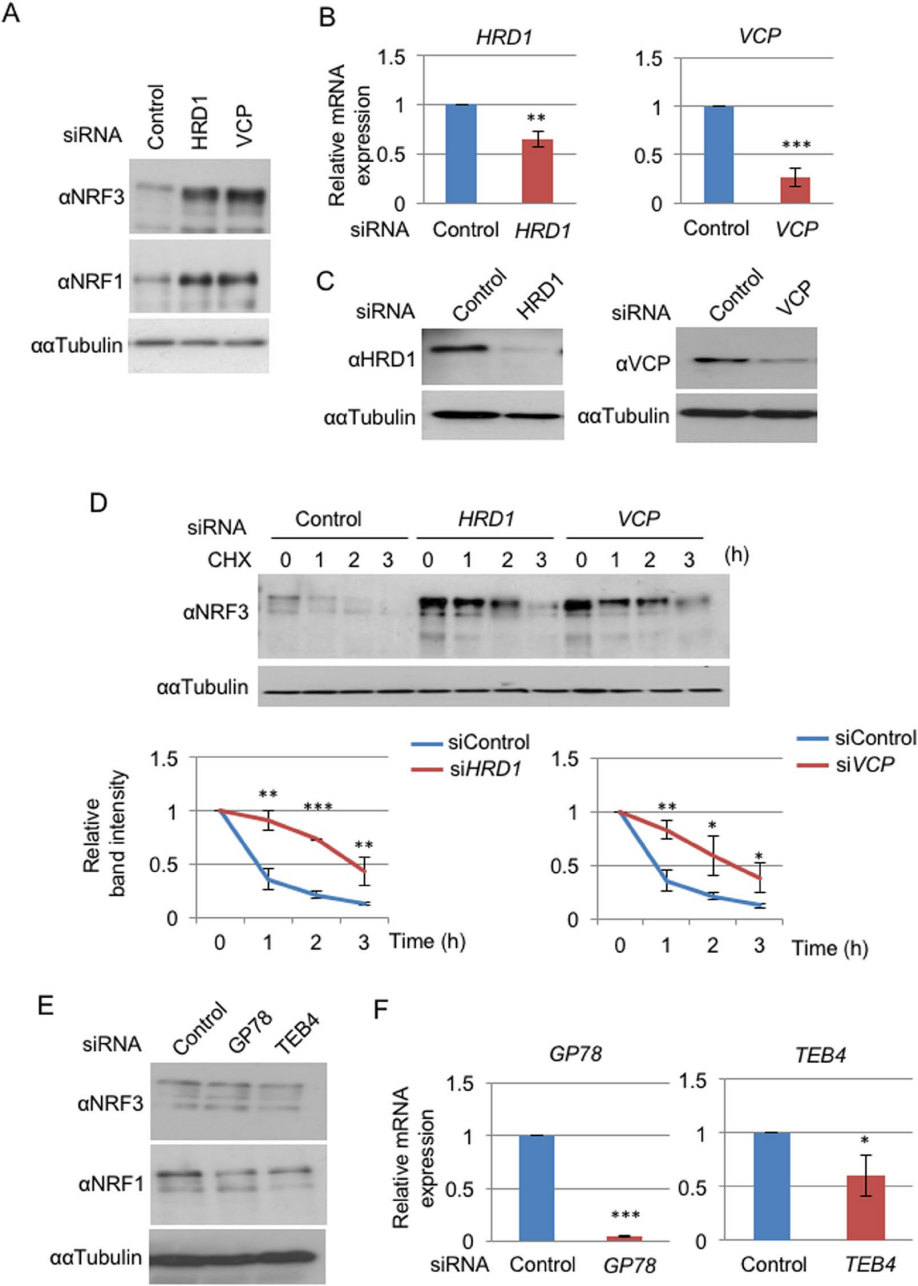
Hrd1 and VCP regulate the cytoplasmic degradation of NRF3. (**A**) The addition of *HRD*1 or *VCP* siRNA stabilized endogenous NRF3 in DLD-1 cells. At 48 hr after the siRNA transfection, the whole-cell extracts were prepared and analyzed by immunoblotting with anti-NRF3 and anti-NRF1 antibodies. α-Tubulin was used as an internal control. (**B,C**) The knockdown efficiency of *HRD1* and *VCP* siRNA was determined by quantitative real-time PCR (qRT-PCR) analysis and immunoblot analysis with anti- HRD1 and anti-VCP antibodies. The values of the qRT-PCR analysis in (**B**) were normalized to the 18S rRNA data. (**D**) The knockdown of *HRD1* or *VCP* inhibited NRF3 degradation in the cycloheximide chase experiment. The immunoblot analysis was performed with the anti-NRF3 antibody. α-Tubulin was used as an internal control. The graphs depict the quantified band intensities of NRF3, normalized to that of α-Tubulin. (**E**) The addition of *GP78* or *TEB4* siRNA did not stabilize the endogenous NRF3 in DLD-1 cells. The experiment was done as described in the legend of Fig. 1A. Error bars (**B,D** and **F**) represent data from three independent experiments (mean ± standard deviation). The two-tailed Student’s t-test was used for the statistical analysis. *P < 0.05, **P < 0.01 and ***P < 0.001 compared to the Control data.

To confirm this hypothesis, we first investigated the effects of *HRD1* or *VCP* knockdown on the stability of endogenous NRF3 in human colon adenocarcinoma DLD-1 cells. *HRD1* or *VCP* siRNA was transfected into the cells, and whole cell extracts were prepared and subjected to immunoblot analysis. *HRD1* or *VCP* knockdown markedly stabilizes the endogenous NRF3 as well as the NRF1 in DLD-1 cells (Fig. 1A). We also verified by real-time quantitative PCR (qRT-PCR) analysis (Fig. 1B) and immunoblot analysis (Fig. 1C) that each siRNA significantly reduces the mRNA and protein expression levels of its respective target, HRD1 or VCP. HRD1- and VCP-mediated degradation of NRF3 was also observed in HCT116 cells by performing similar experiments (Figure S1A and B). Furthermore, a cycloheximide (CHX) chase experiment clearly indicated that the *HRD1* or *VCP* knockdown significantly stabilizes NRF3 (Fig. 1D). Alternatively, we found that the single knockdown of other ERAD-related ubiquitin ligases, *GP78* and *TEB4*, does not stabilize NRF3 in DLD-1 cells (Fig. 1E and F). These results indicate that NRF3 undergoes cytoplasmic degradation via HRD1 and VCP under physiological conditions.

### β-TRCP promotes nuclear degradation of NRF3

**Figure 2:**
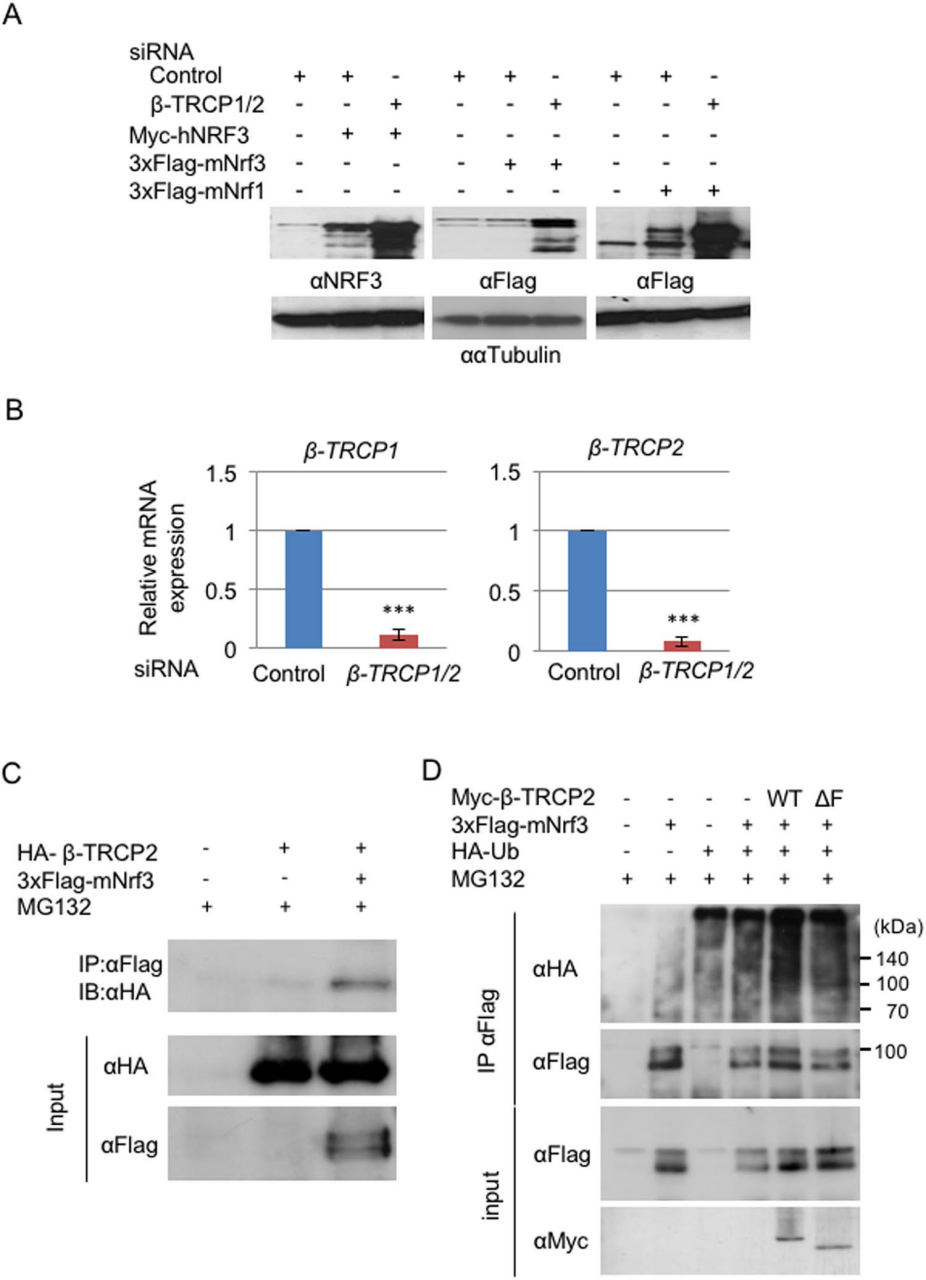
β-TRCP regulates the ubiquitin-mediated degradation of NRF3. (**A**) HeLa cells were transfected with Myc-hNRF3, 3× Flag-mNrf3 or 3× Flag-mNrf1 (as a positive control) expression vectors at 24 hr after two rounds of transfection with the Control or *β-TRCP1/2* siRNA (simultaneous knockdown of both *β-TRCP1* and *β-TRCP2*). At 24 hr after the last transfection, whole-cell extracts from the cells were subjected to immunoblot analysis with anti-NRF3 and anti-Flag antibodies. α-Tubulin was used as an internal control. (**B**) The knockdown efficiency of *β-TRCP1/2* siRNA was determined by real-time quantitative PCR analysis. The values were normalized to 18S rRNA data. Error bars represent data from three independent experiments (mean ± standard deviation). The two-tailed Student’s t-test was used for the statistical analysis. ***P < 0.001 compared to the Control data. (**C**) Physical association between NRF3 and β-TRCP. 3xFlag-mNrf3 and HA-β-TRCP2 were transiently expressed in COS7 cells. The immunoprecipitation was conducted using the anti-Flag antibody, followed by immunoblot analysis using the anti-HA antibody. (**D**) β-TRCP-mediated polyubiquitination of NRF3 in HCT116 cells. The cells were transfected with 3xFlag-mNrf3, HA-ubiquitin (Ub), and the Myc-β-TRCP2 wild type (WT) or the ∆F-box mutant (∆F). The 3xFlag-hNRF3 was immunoprecipitated (IP) with the anti-Flag antibody, and its polyubiquitination was detected by immunoblot analysis with the anti-HA antibody.

We next examined whether β-TRCP, as a cofactor of SKP1, exerts NRF3 degradation in the nucleus because β-TRCP promotes the proteasomal degradation of the NRF3-related factor NRF1^24^. Considering the functional redundancy between β-TRCP1 and β-TRCP2, we simultaneously knocked down both factors by siRNA in all subsequent experiments (β-TRCP1/2). We first investigated the effects of *β-TRCP* siRNA on transiently overexpressed NRF3 in HeLa cells. The *β-TRCP* knockdown markedly stabilizes Myc-tagged human NRF3 (Myc-hNRF3) and 3xFlag-tagged mouse Nrf3 (3xFlag-mNrf3) in HeLa cells, as well as 3xFlag-tagged mouse Nrf1 (3xFlag-mNrf1), which was a positive control (Fig. 2A). We confirmed the significant knockdown of *β-TRCP1* and *β-TRCP2* mRNA in HeLa cells by qRT-PCR analysis (Fig. 2B). Immunoprecipitation experiments revealed a physical association between 3xFlag-mNrf3 and HA-β-TRCP (Fig. 2C). Finally, a ubiquitination assay using cultured cells revealed marked ubiquitination of 3xFlag-mNrf3 in the presence of wild-type β-TRCP2 but not in the presence of the ΔF-box mutant (Fig. 2D, WT and ΔF). This result suggests that β-TRCP promotes the nuclear degradation of NRF3 by its polyubiquitination.

**Figure 3:**
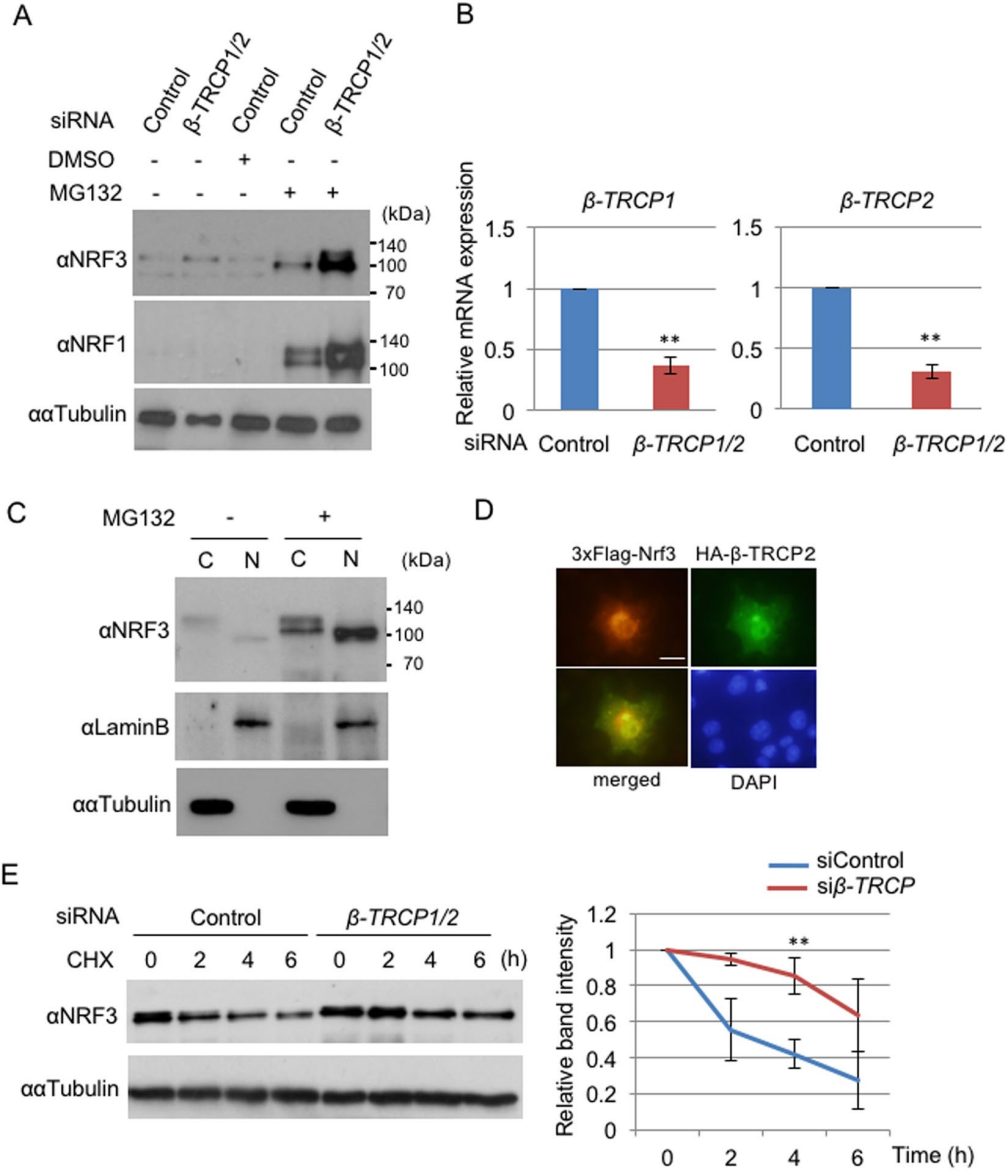
β-TRCP modulates the nuclear degradation of NRF3. (**A**) Endogenous NRF3 is susceptible to the β-TRCP-mediated proteasomal degradation in the nucleus of DLD-1 cells. The cells were transfected with the Control or *β*-*TRCP1/2* siRNA. At 48 hr after transfection, the cells were subjected to two processes: (1) their whole cell extracts were prepared for immunoblot analysis with anti-NRF3 and anti-NRF1 antibodies, and (2) the cells were further treated with DMSO or MG132 (10 µM) for 6 hr, followed by a similar immunoblot analysis. (**B**) The knockdown efficiency of siRNA for *β-TRCP1/2* was determined by qRT-PCR analysis. (**C**) MG132 treatment promoted the nuclear translocation of NRF3 in DLD-1 cells. The cytoplasmic and nuclear fractions of DLD-1 cells treated with MG132 for 6 hr were subjected to immunoblot analysis with the anti-NRF3 antibody. Lamin B and α-Tubulin were utilized as the nuclear and cytoplasmic markers, respectively. (**D**) Nuclear colocalization of 3xFlag-mNrf3 (red) and HA-β-TRCP (green) after MG132 treatment in COS7 cells visualized by immunostaining. The nuclei were stained with DAPI (bar = 20 μm). (**E**) *β-TRCP1/2* siRNA stabilized the endogenous NRF3 in DLD-1 cells in a cycloheximide (CHX) chase experiment. After the siRNA transfection, the cells were treated with MG132 (10 µM) for 6 hr, followed by treatment with cycloheximide. The immunoblot analysis was performed with the anti-NRF3 antibody. α-Tubulin was used as an internal control. The graph (**E**) depicts the quantified band intensities of NRF3. The values were normalized with α-Tubulin. The error bars (**B** and **E**) represent data from three independent experiments (mean ± standard deviation). The two-tailed Student’s t-test was used for the statistical analysis. **P < 0.01 compared to the Control data.

**Figure 4:**
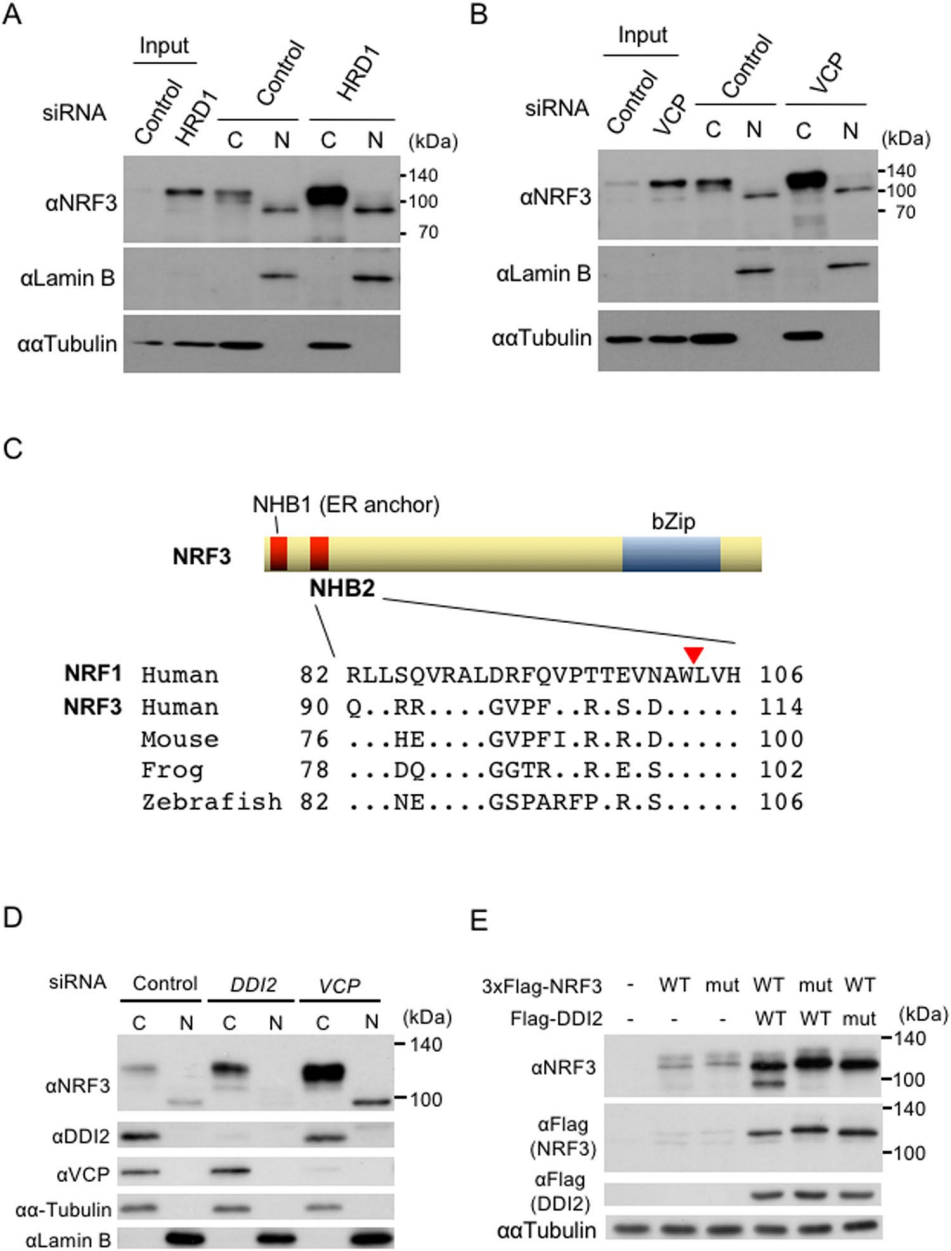
The nuclear translocation of NRF3 requires the aspartic protease DDI2, but not inhibition of HRD1 or VCP. (**A** and **B**) *HRD1* or *VCP* knockdown did not promote the nuclear translocation of NRF3. The DLD-1 cells were transfected with Control and *HRD1* siRNA. At 48 hr after transfection, the cytoplasmic and nuclear fractions were extracted from the cells and subjected to immunoblot analysis with anti-NRF3 antibody. Lamin B and α-Tubulin were utilized as the nuclear and cytoplasmic markers, respectively. (**C**) Sequence alignment of the NHB2 domain of NRF1 and NRF3. The NRF1 processing site (a red triangle)^27^ is highly conserved in NRF3 among several species. (**D**) *DDI2* knockdown substantially abolishes the nuclear translocation of the endogenous NRF3 in DLD-1 cells. After transfection with the indicated siRNA, the cells were fractionated into the cytoplasmic (**C**) and nuclear extracts (N), followed by an immunoblot analysis using the indicated antibodies. As a positive control, a similar experiment using *VCP* siRNA was performed. (**E**) DDI2 cleaves the N-terminal 3xFlag-fused hNRF3. The cells were transfected into HeLa cells with the indicated plasmids, and whole-cell extracts from the cells were subjected to immunoblot analysis with the indicated antibodies. 3xFlag-hNRF3 WL111AA (mut) is the mutant of a putative cleavage site in NRF3 that corresponds to the same site in NRF1^27^, and DDI2 D252N (mut) is the protease dead mutant^28^.

We further examined the effects of β-TRCP on endogenous NRF3 stability in DLD-1 cells (Fig. 3A and B). Unexpectedly, *β-TRCP1/2* siRNA did not promote the accumulation of NRF3 in the cells, although it significantly repressed the *β-TRCP* expression. We assumed that this result is due to different cellular localizations of NRF3 and β-TRCP. NRF3 is mainly localized in the endoplasmic reticulum (ER) under physiological conditions^13^, while β-TRCP mediates the proteasomal degradation of NRF3-related factor NRF1 in the nucleus^24^. In this regard, we found that the proteasome inhibitor MG132 promotes the nuclear entry of endogenous NRF3 (Fig. 3C. The different molecular size of the NRF3 protein in the cytoplasm and nucleus is examined in Fig. 4). In addition, the MG132 treatment caused the predominant nuclear colocalization of 3xFlag-mNrf3 and HA-tagged β-TRCP in the transient overexpression experiment (Fig. 3D). We then performed an experiment similar to that in Fig. 3A with the MG132 treatment (Fig. 3A, MG132+). Consequently, we successfully determined that *β-TRCP* siRNA enhances the stabilization of endogenous NRF3 in the nucleus. A similar result was observed using HCT116 cells (Figure S1C and D). We further validated these results by performing a cycloheximide (CHX) chase experiment that showed that *β-TRCP* knockdown stabilizes endogenous NRF3 (Fig. 3E). Overall, these data clearly demonstrate that β-TRCP mediates NRF3 degradation in the nucleus, and furthermore that β-TRCP is not involved in the process of the nuclear translocation of NRF3.

### The nuclear translocation of NRF3 requires the aspartic protease DDI2, but not inhibition of HRD1 or VCP

A main purpose of this study is to elucidate the molecular basis underlying the nuclear translocation of NRF3 from ER sequestration in cancer cells. We previously identified that the NRF3-related factor NRF2 translocates into the nucleus by repressing a NRF2 degradation mechanism^25,26^. Based on this finding, we inferred that the inhibition of HRD1-VCP-mediated NRF3 degradation might be the natural translocation mechanism. We thus explored the effects of *HRD1* knockdown on the nuclear accumulation of NRF3 in DLD-1 cells (Fig. 4A and B). Unexpectedly, the endogenous NRF3 accumulated in the cytoplasm but not in the nucleus upon treatment with *HRD1* or *VCP* siRNA. Our findings strongly suggest that the nuclear translocation of NRF3 is not due to inhibition of its HRD1- and VCP-mediated degradation.

We discovered the distinct molecular size of endogenous NRF3 in the cytoplasm and nucleus (Figs 3C, 4A and B), so we then changed our strategy to test a new hypothesis: that the nuclear translocation of NRF3 is carried out by its processing. Supporting this speculation, it has been recently reported that NRF3-related factor NRF1 requires processing of its N-terminal homology box 2 (NHB2) domain for its nuclear translocation^27^ and furthermore that the aspartyl proteases DDI2 and DDI1 are involved in the nuclear entry of NRF1 and the *C. elegans* homolog SKN-1A, respectively^28,29^. The NHB2 domain is highly conserved between NRF1 and NRF3 (Fig. 4C), indicating that DDI2 may cleave NRF3 as well. To confirm this hypothesis, we investigated the effects of *DDI2* knockdown on endogenous NRF3 protein levels in the nucleus by immunoblot analysis using cell fractionated extracts (Fig. 4D). Again, this experiment showed distinct molecular sizes of NRF3 proteins in the cytoplasm and nucleus (approximately 130 and 100 kDa, respectively). As expected, *DDI2* siRNA reduced the nuclear NRF3 protein levels and also enhanced its cytoplasmic levels (Fig. 4D), indicating that DDI2 plays a role in the nuclear entry of NRF3.

We further examined whether DDI2 processes 3xFlag-hNRF3 in the transient expression system using HeLa cells (Fig. 4E). For a negative control, we generated an expression vector of 3xFlag-hNRF3 WL111AA that harbors an alanine mutation in the Trp-Leu motif in the NHB2 domain, which corresponds to the processing site in NRF1^27^. The transfection of 3xFlag-hNRF3 alone generated a 120-kDa product that was recognized by both anti-NRF3 and anti-Flag antibodies, implying that it is unprocessed full-length 3xFlag-hNRF3. Significantly, the coexpression of DDI2 caused a product of lower molecular weight (approximately 100 kDa). The shorter product was recognized only by the anti-NRF3 antibody, suggesting that it is a 3xFlag-hNRF3 protein that is lacking the N-terminal Flag epitope, presumably due to DDI2 cleavage. Moreover, we could not find the shorter form by coexpression of either the 3xFlag-hNRF3 WL111AA or protease-dead DDI2 mutants (DDI2 D252N). We thus conclude that DDI2 is required for the N-terminal processing and, thereby, nuclear translocation of NRF3.

### NRF3 modulates the gene expression of the cell cycle regulator UHMK1

**Figure 5:**
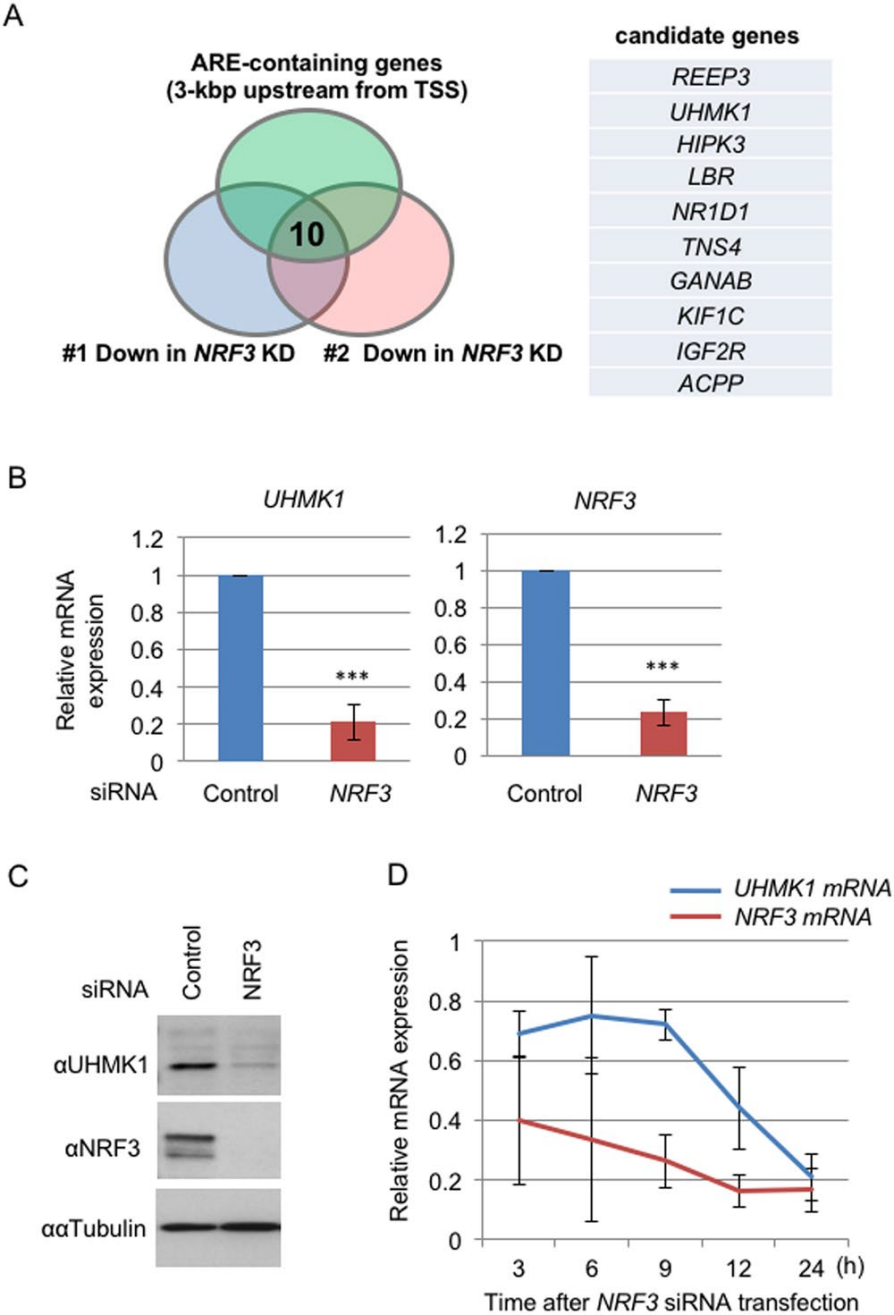
Identification of the *UHMK1* gene as a target of NRF3. (**A**) Venn diagram combining two independent sets of microarray data of *NRF3* siRNA-transfected DLD-1 cells (#1 and #2) and a list of genes that possess the species-conserved AREs within the region that is 3 kb upstream from the transcriptional start site (TSS). Ten candidate genes from this analysis are shown. (**B** and **C**) *NRF3* knockdown significantly reduces mRNA and protein levels of UHMK1 in DLD-1 cells. At 48 hr after transfection with Control or *NRF3* siRNA, the mRNA expression levels of *UHMK1* and *NRF3* were determined by qRT-PCR analysis. The values were normalized to 18S rRNA data (**B**). Immunoblotting of the whole-cell extracts with the anti-NRF3 and anti-UHMK1 antibodies was performed (**C**). α-Tubulin was used as an internal control. (**D**) A time-course study of *UHMK1* mRNA expression after the *NRF3* knockdown. The DLD-1 cells were transfected with Control or *NRF3* siRNA, after which the mRNA of the cells was extracted at the indicated times and a qRT-PCR analysis was performed. The error bars (**B**,**D**) represent data from three independent experiments (mean ± standard deviation). The two-tailed Student’s t-test was used for the statistical analysis. ***P < 0.001 compared to the Control data.

Finally, we tackled the next issue regarding the NRF3 target gene(s) in cancer cells. To this end, microarray analyses were performed to identify the genes whose expression was reduced upon siRNA-mediated *NRF3* knockdown (Fig. 5A). In addition, we listed the genes possessing species-conserved ARE sequences in the 3-kbp region that is upstream from their transcriptional start sites. Computationally combining these data highlighted 10 genes as putative NRF3 target genes. Among candidate genes, we focused on the *UHMK1* (*KIS*) gene because UHMK1 has been reported to regulate cell cycle progression by phosphorylating the tumor suppressor p27Kip1 (cyclin-dependent kinase (CDK) inhibitor)^22,23^. Together, qRT-PCR and immunoblot analyses confirmed that *NRF3* knockdown reduces the mRNA and protein expression of the *UHMK1* gene, respectively (Fig. 5B and C). The result was consistently observed in DLD-1 and HCT116 cells using additional *NRF3* siRNA (NRF3(A)) (Figures S2 and S3).

To exploit whether NRF3 directly mediates the *UHMK1* expression through the ARE sequence in its promoter, we performed a chromatin immunoprecipitation (ChIP) analysis. However, due to unknown reasons, this experiment did not succeed. Alternatively, we performed a time-course study to examine the *UHMK1* expression upon siRNA-mediated *NRF3* knockdown (Fig. 5D). After the transfection of *NRF3* siRNA into DLD-1 cells, the *NRF3* and *UHMK1* mRNA were monitored over time by qRT-PCR analysis at the indicated time points. We found that the curve that exhibits the reduction of *UHMK1* mRNA expression is slightly delayed compared with that of the *NRF3* mRNA, implying that NRF3 is an upstream regulator of *UHMK1* gene expression. These results clearly demonstrate that NRF3 regulates *UHMK1* expression in colon cancer cells.

### NRF3 promotes the proliferation of colon cancer cells

**Figure 6:**
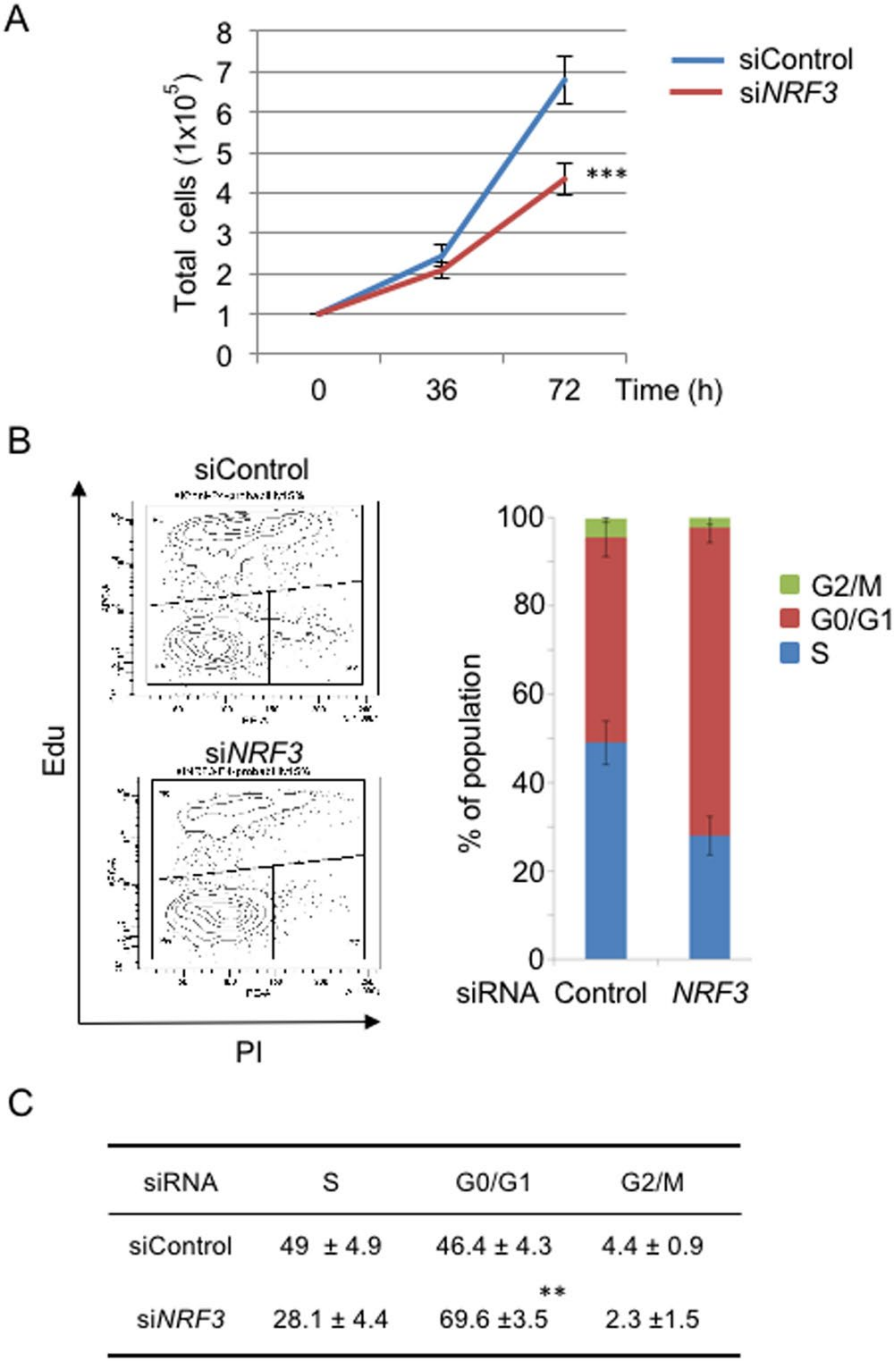
NRF3 promotes the proliferation of colon cancer cells. (**A**) *NRF3* knockdown significantly reduced the proliferation of DLD-1 cells. The cells were transfected with Control or *NRF3* siRNA. At 36 and 72 hr after transfection, the cell numbers were counted using a hemocytometer. The initial cell numbers at the time of transfection were 1 × 10^5^. (**B** and **C**) *NRF3* knockdown significantly arrested DLD-1 cells in the G0/G1 phase. At 48 hr after transfection with the Control or *NRF3* siRNA, the cells were subjected to FACS analysis to determine the fraction of their populations in different cell cycle stages (G0/G1, S and G2/M). The representative data from three independent experiments are shown (**B**, left). The percentages of the cell population in each phase are shown as the mean ± standard deviation (**C**). The error bars (**A**,**B**) represent data from three independent experiments (mean ± standard deviation). The two-tailed Student’s t-test was used for the statistical analysis. ***P < 0.001 (**A**) and **P < 0.01 (**C**) compared to the Control data.

Given that UHMK1 induces the proliferation and cell cycle progression of cancer cells^22,23^, our findings gave rise to the next important question: whether NRF3 knockdown reduces cell proliferation. To address this question, we examined the effects of *NRF3* siRNA on the proliferation of DLD-1 cells by counting cell numbers (Fig. 6A). As expected, the *NRF3* knockdown significantly reduced the cell proliferation. A cell cycle analysis using a flow cytometer (FACS) demonstrated that the *NRF3* knockdown significantly causes cell cycle arrest (G0/G1) and a reduction of the G2/M and S populations of DLD-1 cells (Fig. 6B and C). Moreover, we examined whether reduction of cell proliferation by *NRF3* knockdown is due to suppression of the UHMK1 expression (Figure S4). Consistently, two-independent *UHMK1* siRNAs significantly reduced proliferation of DLD-1 cell as well as *NRF3* siRNA did. Finally, we further investigated an involvement of NRF3-related factor NRF1 or NRF2 in the *UHMK1* gene expression. Surprisingly, *NRF1* or *NRF2* knockdown also reduces the gene expression in DLD-1 cells (Figure S5). It suggests the presence of cooperative regulation of *UHMK1* gene expression by these NRF3-related factors. Altogether, these results clearly demonstrate that NRF3 promotes colon cancer cell proliferation by activating *UHMK1* gene expression.

## Discussion

**Figure 7:**
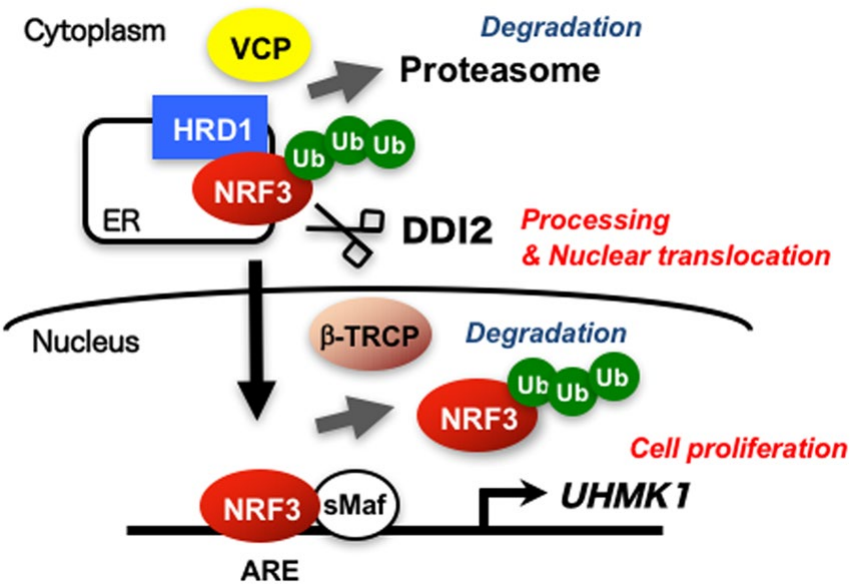
Schematic model of multiple regulation of the biological function of the transcription factor NRF3. Under normal conditions, NRF3 is degraded by the ERAD ubiquitin ligase HRD1 and VCP in the cytoplasm. DDI2 promotes the nuclear entry of NRF3 by its processing. In the nucleus, NRF3 activates the expression of the *UHMK1* gene for cell proliferation. Alternatively, the β-TRCP-based E3 ubiquitin ligase suppresses the NRF3 function by mediating its nuclear degradation.

In this study, we investigated the molecular basis behind NRF3 activation, i.e., its nuclear translocation and its biological function in cancer cells. Our schematic model in Fig. 7 summarizes the regulatory mechanisms of NRF3 function that are tightly coupled to several protein degradation and processing systems. Our discovery that NRF3 promotes cancer cell proliferation by inducing the UHMK1 gene expression is a first report of physiological role of NRF3 in cancer. These observations will help us understand the molecular basis of the physiological roles of NRF3 in cancer that we will report in the near future.

We found that the DDI2 is required for the nuclear translocation of NRF3. This result is consistent with recent reports that DDI2 and the *C. elegans* homolog DDI1 elicit the nuclear translocation of NRF3-related factor NRF1 and its homolog SKN-1A, respectively^28,29^. DDI2 is an aspartic protease, which is highly conserved throughout eukaryotes^30^. We also determined that the nuclear entry of NRF3 does not occur by the inhibition of the HRD1-mediated cytoplasmic degradation of NRF3 (Fig. 4A). This pathway is in contrast to NRF2, in which nuclear entry is triggered by suppressing NRF2 degradation in response to oxidative stress^25,26^. Thus, these observations strongly suggest the difference of activation mechanism and thereby biological roles between NRF2 and NRF3.

The cleaved site in the NHB2 domain of NRF1 is highly conserved in NRF3 (Fig. 4C). Mutation into the retroviral protease-like (RVP) domain in DDI2 or the putative cleavage sites of NRF3 substantially attenuates the cleavage and nuclear entry of NRF3 (Fig. 4E). These results suggest that DDI2 is required to cleave NRF3 via the RVP domain, similar to the case of NRF1. We previously found that a degron in NRF1 for HRD1-driven degradation is located in 31–81 amino acid residues that are adjacent to the N-terminus of the NHB2 domains^24^. Thus, it is likely that the DDI2 cleavage stabilizes and liberates NRF1 from the ER by removing the degron and the NHB1 domain, which functions as an ER-anchor domain. We further infer that this regulation of NRF3 is similar to that of NRF1 because of conservation of the amino acid sequences in their NHB1 and NHB2 domains.

Identification of the NRF3-DDI2 relationship gave rise to the next question: how is the processing of NRF3 by DDI2 regulated? We consider that this question is equivalent to the crucial issue of how NRF3 is activated to gain entry into the nucleus. To answer this question, we presumably need the comprehensive identification and functional examination of the regulatory factors in this system. Recently, genome-wide screens have elegantly reported that UBXD8 and NGLY1 are involved in the regulation of NRF1 and its *C. elegans* homolog SKN1^28,29,31^. Given that UBXD8, the ER membrane protein, transfers ubiquitinated ERAD substrates to VCP, it may recognize a ubiquitin chain that is conjugated to NRF3. Our data show that the HRD1-mediated ubiquitination of NRF3 is required for both its proteasomal degradation and its DDI2-mediated processing (Fig. 4A), implying the presence of a regulatory element that can switch from the degradation of NRF3 to its cleavage by DDI2. NGLY1 is a deglycosylation enzyme for that proteasomal degradation of ERAD substrates. It has also been consistently reported that NRF1 is deglycosylated before its cleavage by DDI2^27,28^. Finally, we also have no answer to another crucial question regarding the NRF3 activation signal/stimuli. The identification of the NRF3 target genes, including *UHMK1*, should provide us clues to clarify this question.

Our study discovered that NRF3 and NRF1 are under the control of the same regulatory systems, i.e., HRD-VCP, β-TRCP and DDI2. This result is very reasonable because *NRF3* and *NRF1* are believed to be derived from the common ancestral *CNC* gene (*Drosophila*). Thus, it further suggests that NRF3 and NRF1 share common biological functions. Nevertheless, gene-targeting experiments in mice suggest distinct physiological functions in Nrf3 and Nrf1. *Nrf3* knockout mice do not exhibit apparent abnormalities under normal conditions^5,6^, while *Nrf1* knockout mice show embryonic lethality due to anemia^32^. Their functional differences might be due to a difference in their activation mechanisms. For example, we preliminarily found that *NRF3* knockdown significantly induces NRF1 protein accumulation, which rescues loss of NRF3 function in human colon cancer HCT116 cells, although *NRF1* knockdown does not induce NRF3 protein (data not shown). This compensatory mechanism by NRF1 might be a reason why *Nrf3* knockout mice do not exhibit severe abnormalities. Accordingly, we consider that NRF3 and NRF1 are activated at least in part by distinct biological pathways. The CNC family proteins in higher eukaryotes might acquire diversity and complexity during the evolutionary progress from the common ancestral *CNC* gene in *Drosophila*.

We discovered that NRF3 causes cell proliferation by inducing *UHMK1* gene expression. It is quite reasonable that the UHMK1 kinase promotes cell proliferation by repressing the cyclin-dependent kinase (CDK) inhibitor p27Kip1 through phosphorylation and then activating CDK in G1 phase^22,23,33^. The time-course study of the *NRF3* and *UHMK1* gene expression upon *NRF3* knockdown in DLD-1 cells fairly suggests that NRF3 is an upstream activator of *UHMK1* gene (Fig. 5D). While *NRF3* siRNA reduced half cell proliferation of DLD-1 cells (Fig. 6A), we have recently found that it completely diminishes cell proliferation of HCT116 cells (data not shown). A difference between these cell lines is at least the tumor suppressor gene *TP53* status, *i.e*. HCT116 and DLD-1 cells possess wild-type and mutant *TP53* gene, respectively. This result implies that NRF3 regulates cell growth through multiple cascades including the UHMK1 and p53 pathways. These current observations regarding the physiological roles of NRF3 in cancer will be published as a next project in the near future.

This study further proposes one attractive idea: develop a new anticancer therapeutic strategy by targeting DDI2. The knockdown of *DDI2* reduces the nuclear entry of NRF3, presumably reducing NRF3-mediated cell growth. Accordingly, it is possible that DDI2 inhibitors work as anticancer drugs by repressing the NRF3 activity. Intriguingly, the retroviral protease-like (RVP) domain of yeast homolog Ddi1p structurally exhibits a similar fold to those of HIV protease domains^34^. Thus, HIV therapeutic drugs that target the HIV protease might be repositioned as anticancer drugs that suppress the peptidase activity of DDI2.

## Methods

### Preparation of a monoclonal antibody against NRF3

A monoclonal NRF3 antibody (#9408) raised against human NRF3 (amino acids 364-415) was generated as described previously^35^.

### Antibodies

The antibodies utilized in the current immunoblot analysis were anti-NRF3 (#9408), anti-FLAG (M2; Sigma), anti-α-Tubulin (DM1A; Sigma), anti-Lamin B (Invitrogen), anti-Nrf1 (D5B10; Cell Signaling Technology), anti-HRD1 (D302A; Cell Signaling Technology), anti-VCP (H-120; Santa Cruz), anti-HA (Y-11; Santa Cruz, 3F-10; Roche), anti-Myc (A-14; Santa Cruz), anti-DDI2 (A304-629; Betyl Laboratories) and anti-KIS1 (UHMK1) (a kind gift from Alexandre Maucuer, Universite Pierre et Marie Curie).

### Plasmid

The 3xFlag-mNrf3 plasmid was generated by subcloning the PCR-amplified mouse Nrf3 cDNA into the p3xFLAG-CMV^TM^ 10 vector (Sigma). Myc-hNRF3 was kindly provided by Yiguo Zhang (Chongqing University)^13^. PCR-amplified hNRF3 cDNA was subcloned into the p3xFLAG-CMV^TM^ 10 vector (3xFlag-hNRF3). Site-directed mutagenesis of 3xFlag-hNRF3 encoding the N-terminal putative processing site (3xFlag-hNRF3 WL111AA) was performed by a PCR-based method using the indicated primers. Forward: 5′-CACCAGCGTGGATGCAGCAGCTGTGCACAGCGTGGCTGC-3′, Reverse: 5′-GCAGCCACGCTGTGCACAGCTGCTGCATCCACGCTGGTG-3′. The DDI2 and DDI2 D252N mutant vectors were kindly provided by Shigeo Murata^28^. The generation of 3xFlag-Nrf1, HA–β-TRCP2, HA-ubiquitin, Myc-tagged β-TRCP2 and ΔF-box β-TRCP2 plasmids were described previously^24^.

### Cell culture and transfection

DLD-1, HCT116, HeLa and COS7 cells were cultured in Dulbecco’s modified Eagle’s medium (DMEM) (Wako) supplemented with 10% fetal bovine serum (FBS) (Nichirei), 40 μg/ml streptomycin, and 40 units/ml penicillin (Life Technology). The transfection of the plasmid DNA and short interfering RNA (siRNA) was performed using Lipofectamine 2000 or polyethylenimine (PEI) and RNAiMAX (Invitrogen), respectively.

### Immunoblot analysis

To prepare whole cell extracts, the cells were lysed with SDS sample buffer (50 mM Tris-HCl [pH 6.8], 10% glycerol and 1% SDS). The protein quantities in cell extracts were measured with a bicinchoninic acid (BCA) kit (Thermo). The proteins were separated by sodium dodecyl sulfate-polyacrylamide gel electrophoresis (SDS-PAGE) and transferred to PVDF membranes (Immobilon-P transfer membrane, EMD Millipore corporation, Billerica, USA). Immunoblot analysis was performed as described previously^24^. The membranes was blocked with Blocking one (Nacalal Tesque) or TBS-T (20 mM Tris-HCl [pH 7.6], 137 mM NaCl, 0.1% Tween20) containing 5% skim milk and 10% goat serum (Invitrogen) at 4 °C for overnight.

### Cycloheximide chase experiments

DLD-1 cells were transfected with the indicated siRNA. At 48 hr after transfection, the cells were treated with 20 μg/ml cycloheximide (CHX), and the whole cell extracts were prepared at the indicated time points. The immunoblot analysis was conducted with the indicated antibodies. In the case of β-TRCP-related experiments, the cells were treated with 10 µM MG132 for 6 hr at 48 hr after siRNA transfection to promote the nuclear translocation of NRF3, then washed twice with phosphate-buffered saline (PBS) and treated with 20 µg/ml cycloheximide.

### Cell fractionation

At 48 hr after transfection of the indicated siRNA into DLD-1 cells, cell fractionation was performed as described previously^24^.

### Immunoprecipitation

COS7 cells were transfected with 3хFlag-mNrf3 or HA–β-TRCP2 plasmids. At 24 hr after transfection, the cells were treated with MG132 (10 µM) for 6 hr. The preparation of whole cell extracts and immunoprecipitation were conducted as described previously^24^.

### Immunocytochemical staining

COS7 cells were transfected with 3хFlag-mNrf3 and/or HA-β-TRCP2 plasmids. At 24 hr after transfection, the cells were treated with MG132 (10 µM) for 6 hr. The staining was conducted as described previously^24^.

### The ubiquitination assay

HCT116 cells were transfected with 3хFlag-mNrf3 and HA-ubiquitin, along with the wild-type Myc-β-TRCP2 or the ΔF-box β-TRCP2 mutant. At 24 hr after transfection, the cells were treated with MG132 (10 µM) for 6 hr and the whole cell extracts were prepared with lysis buffer (10 mM Tris-HCl [pH 7.5], 150 mM NaCl, 1% SDS, 1х protease inhibitor cocktail (Nacalal Tesque), 10 µM MG132, and 10 mM N-ethylmaleimide (NEM)). The cell extracts were then boiled and sonicated. After centrifugation at 18,000 x g for 15 min at 4 °C, the supernatants were diluted with dilution buffer (10 mM Tris-HCl [pH 7.5], 150 mM NaCl, 1% Triton X-100, 1 х protease inhibitor cocktail, 10 µM MG132, and 10 mM NEM) to reduce the SDS concentration to less than 0.03%, at which point they were incubated with anti-Flag antibody and protein G Sepharose beads (GE Healthcare) at 4 °C overnight. The immunocomplexes were washed three times with dilution buffer and eluted by boiling in SDS sample buffer. The ubiquitinated Nrf3 was visualized by immunoblot analysis using the anti-HA antibody.

### Microarray Analysis

Total RNA was processed with the Ambion WT Expression Kit (Affymetrix) according to the manufacturers’ instructions. cRNA was then fragmented, labelled, and hybridized to the Affymetrix Human Gene 1.0 ST Arrays using the Gene Chip WT Terminal Labeling and Hybridization Kit (Affymetrix). GeneChip fluidics station 450 was used for processing of the arrays and fluorescent signals were detected with the GeneChip scanner 3000-7 G. Images were analyzed with the GeneChip operating software (Affymetrix). Finally, Expression console and Transcription analysis console (Affymetrix) were used to identify the genes whose expression was reduced upon siRNA-mediated *NRF3* knockdown (fold change ≧ 1.5). In this study, microarray analysis was performed in duplicate. Data were submitted to Gene Expression Omnibus database (accession number GSE99080).

### siRNA knockdown experiment

The DLD-1 and HCT116 cells were cultured for 16 hr in the medium without antibiotics. The cells were then transfected with 40 nM siRNA using RNAiMAX. At 48 hr after transfection, the cells were utilized for all experiments except cell counting. For FACS and cell counting experiments, the cells were cultured in the medium without antibiotics and transected with 40 nM siRNA by using RNAiMAX without prior incubation. HeLa cells were cultured for 16 hr in the medium without antibiotics. The cells were transfected twice with 40 nM siRNA (at 16 and 40 hr after plating) using RNAiMAX. At 24 hr after the last transfection, the cells were transected with the indicated plasmids and incubated for 24 hr. The sequences of the sense strands of the siRNA duplexes that were employed in the present study are listed in Table 1.

**Table 1:** Sequences of siRNA and primers for qRT-PCR.

Gene	Sense strand sequence (5′-3′)	
*Control*	UUCUCCGAACGUGUCACGUdTdT	
*β-TRCP1/2*	GUGGAAUUUGUGGAACAUCdTdT	
*VCP*	GUAGGGUAUGAUGACAUUGdTdT	
*HRD1*	GGUGUUCUUUGGGCAACUGdTdT	
*NRF3*	CGCAAAUUGGACAUAAUUUdTdT	
*NRF3(A)*	GCAAAGAAGGAAACUCUUAdTdT	
*GP78*	CAUGCAGAAUGUCUCUUAAdTdT	
*NRF1*	GGGAUUCGGUGAAGAUUUGdTdT	
*NRF2*	GUAAGAAGCCAGAUGUUAAdTdT	
*UHMK1*	UACUUUACAUCCUGAUUGCdTdT	
*UHMK1(A)*	UUCAUAUGUGGAAUAACCCdTdT	
*TEB4*	UUAAGAGUGUGCUGCCUAAdTdT	
*DDI2*	GCCAAGUAGUGAUGCUUUAdTdT	
**Gene**	**Forward primer (5′-3′)**	**Reverse primer (5′-3′)**
*NRF3*	CTGACTGGGAAGGCAGAAAAG	TCAGGCTGTGATGAAAGCAA
*NRF1*	TGGAACAGCAGTGGCAAGATCTCA	GGCACTGTACAGGATTTCACTTGC
*NRF2*	TACTCCCAGGTTGCCCACA	CATCTACAAACGGGAATGTCTGC
*UHMK1*	AGAGAAACCATGGGCAGAAG	CAAGCCATGAAACAGCATCT
*HRD1*	TGCAACCACATTTTCCATACCA	GCGATGCACGAAGGACATC
*VCP*	TACCAACCGGCCTGACAT	TGGCAACACGGGACTTCT
*β-TRCP1*	TGCCGAAGTGAAACAAGC	CCTGTGAGAATTCGCTTG
*β-TRCP2*	TCAGTGGCCTACGAGATA	ACACGCTCATCATACTGCA
*GP78*	GGTGCAGCGTAAGGACGAA	GCATCATCTTCAGAACTTTTGTTCA
*TEB4*	TTGTCCTTCCAAGTCCGCCAG	GACTGTGGAGGTGGTGGAGATG
*18S rRNA*	CGCCGCTAGAGGTGAAATTC	CGAACCTCCGACTTTCGTTCT
*β-Actin*	CCAACCGCGAGAAGAT	CCAGAGGCGTACAGGG

### RNA extraction and quantitative real-time PCR (qRT-PCR)

The total RNA was prepared using ISOGEN II (Nippon Gene). One microgram of total RNA was utilized for cDNA synthesis using random hexamer primers (Takara Bio) and the Moloney murine leukemia virus (M-MLV) reverse transcriptase (Invitrogen). Quatitative real-time PCR was conducted using the SYBR Premix Ex Taq II (Takara Bio) and the Thermal Cycler Dice Real Time System II (Takara Bio). The sequences of the primers that were used are listed in Table 1.

### Cell cycle analysis using FACS

The cell cycle analysis was conducted using Click-iT® EdU Flow Cytometry Assay Kits (Invitrogen) according to the manufacturer’s protocol. DLD-1 cells were transfected with the indicated siRNA. At 48 hr after transfection, the cells were treated with EdU (10 μM) for 2 hr at 37 °C and washed twice with 1% BSA in PBS. The cells were fixed by using Click-IT fixative (containing paraformaldehyde) for 15 min at room temperature, followed by washing them twice with 1% BSA and then permeabilizing with P/W (1x Click-iT saponin-based Permeabilization and Wash Reagent) for 15 min at room temperature. After their treatment with Click-iT Reaction Mixtures for 30 min at room temperature in a dark place, the cells were then washed with P/W and stained with Propidium Iodide (PI) buffer for 20 min at 37 °C in a dark place. Finally, the cells were washed twice with P/W and subjected to the cell cycle analysis using FACS.

### Cell counting

DLD-1 cells were plated onto 6-well dishes (1 × 10^5^ cells per well), transfected with the indicated siRNA and cultured for 72 hr. The cells were detached from plates with 0.05% trypsin and gently suspended with ice-cold PBS. The cell counting was performed using a hemocytometer.

### Statistical analysis

The statistical significance of repeated measurements was evaluated using Student’s t-test. All values are represented as the means ± standard deviation for at least three-independent experiments.

### Data availability

All data generated or analyzed during this study are included in this published article and its Supplementary Information files.

## Electronic supplementary material

Supplement figures
