# Supplementary material for: Multiple regulatory mechanisms of the biological function of NRF3 (NFE2L3) control cancer cell proliferation
Source: Sci Rep. 2017 Oct 2;7:12494. doi: 10.1038/s41598-017-12675-y (PMC5624902; doi:10.1038/s41598-017-12675-y)
Supplement: Supplementary file 1 — Supplement figures [file 41598_2017_12675_MOESM1_ESM.pdf]

Supplementary information

Multiple regulatory mechanisms of the biological function of NRF3 (NFE2L3) control cancer cell proliferation

A M Masudul Azad Chowdhury[1], Hiroki Katoh[1], Atsushi Hatanaka[1], Hiroko Iwanari[2], Nanami Nakamura[1], Takao Hamakubo[2], Tohru Natsume[3], Tsuyoshi Waku[1], Akira Kobayashi[1]*

[1]Laboratory for Genetic Code, Graduate School of Life and Medical Sciences, Doshisha University, Kyotanabe, Kyoto, Japan, [2]Department of Quantitative Biology and Medicine, Research Center for Advanced Science and Technology, The University of Tokyo, Tokyo, Japan, [3]National Institutes of Advanced Industrial Science and Technology, Biological Information Research Center (JBIRC), Tokyo, Japan.

Address correspondence to:
Akira Kobayashi
Laboratory for Genetic Code, Graduate School of Life and Medical Sciences,
Doshisha University, 1-3 Tatara Miyakodani, Kyotanabe 610-0394, Japan
Phone: +81-774-65-6273, Fax +81-774-65-6274
E-mail: akobayas@mail.doshisha.ac.jp

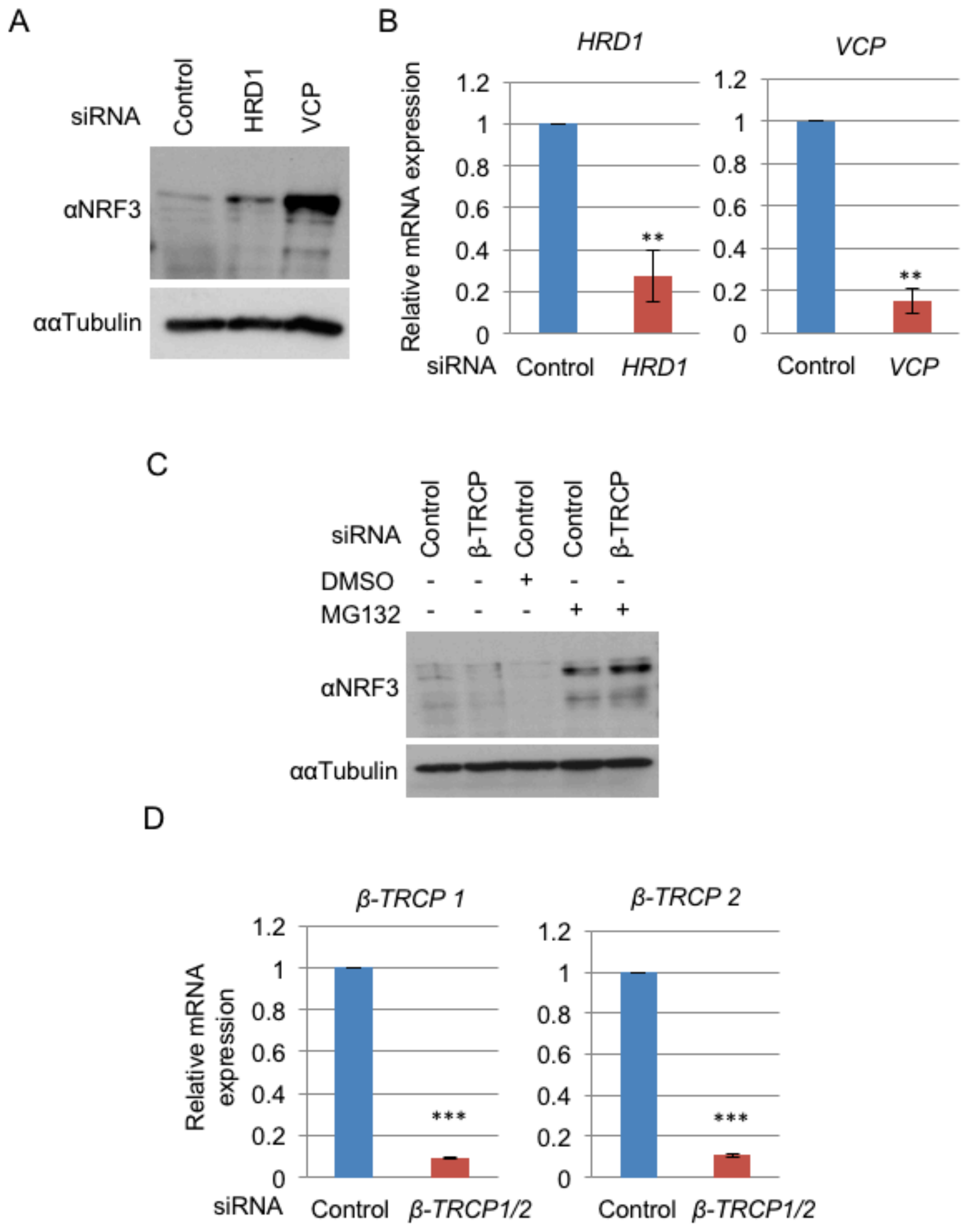

Figure S1. HRD1 and β-TRCP also regulate the NRF3 degradation in HCT116 cells. (A and B) *VCP* and *HRD1* siRNA stabilized the endogenous NRF3 in HCT116 cells (A). The knockdown efficiency of *HRD1* and *VCP* siRNA was determined using qRT-PCR analysis (B). The values were normalized to *β-actin* data. These experiments were performed as described in the legend of Figures 1. (C and D) β-TRCP promotes the degradation of the endogenous NRF3 in HCT116 cells (C). The knockdown efficiencies of *β-TRCP1 and β-TRCP2* siRNA were determined using qRT-PCR analysis (D). The values were normalized to *β-actin* data. These experiments were performed as described in the legend of Figures 3. The two-tailed Student's t-test was used for the statistical analysis. ** $P < 0.01$ and *** $P < 0.001$ compared to the Control data.

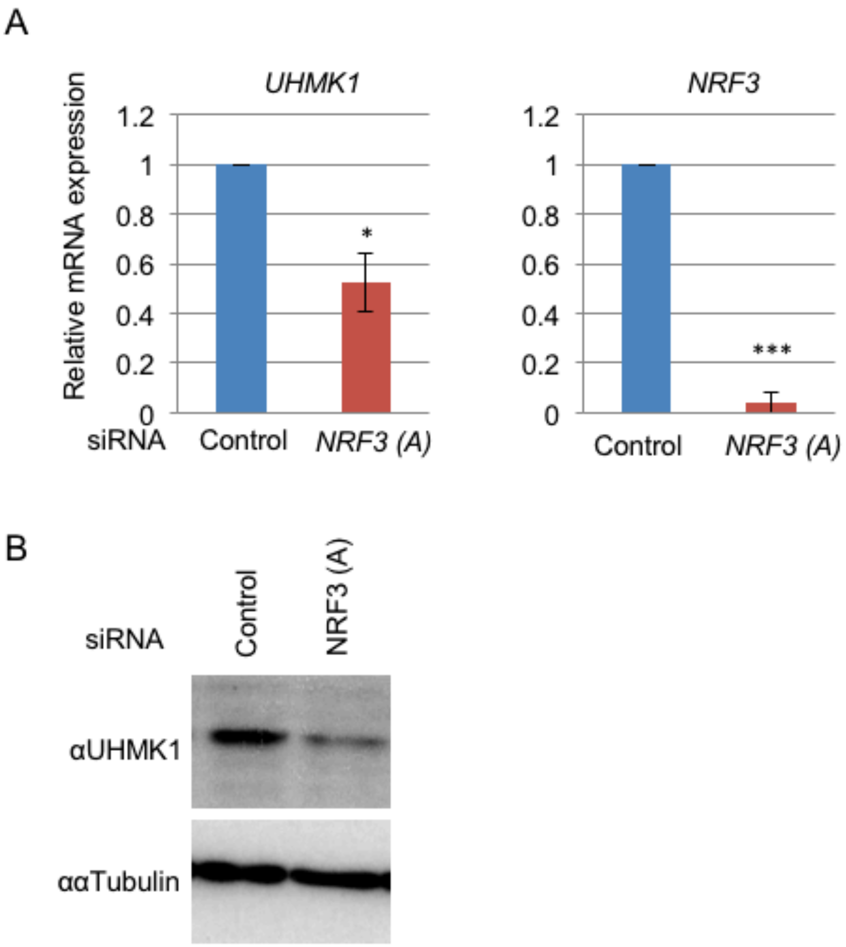

Figure S2. Additional NRF3 siRNA also reduces the UHMK1 expression in DLD-1 cells. (A and B) The effects of additional *NRF3* siRNA (NRF3 (A)) on the mRNA and protein expression levels were determined by qRT-PCR and immunoblot analyses, respectively. The detailed experimental procedure was described in the legend of Figures 5. The error bars (A) represent data from three independent experiments (mean ± standard deviation). The two-tailed Student's t-test was used for the statistical analysis. *$P < 0.05$ and *** $P < 0.001$ compared to the Control data.

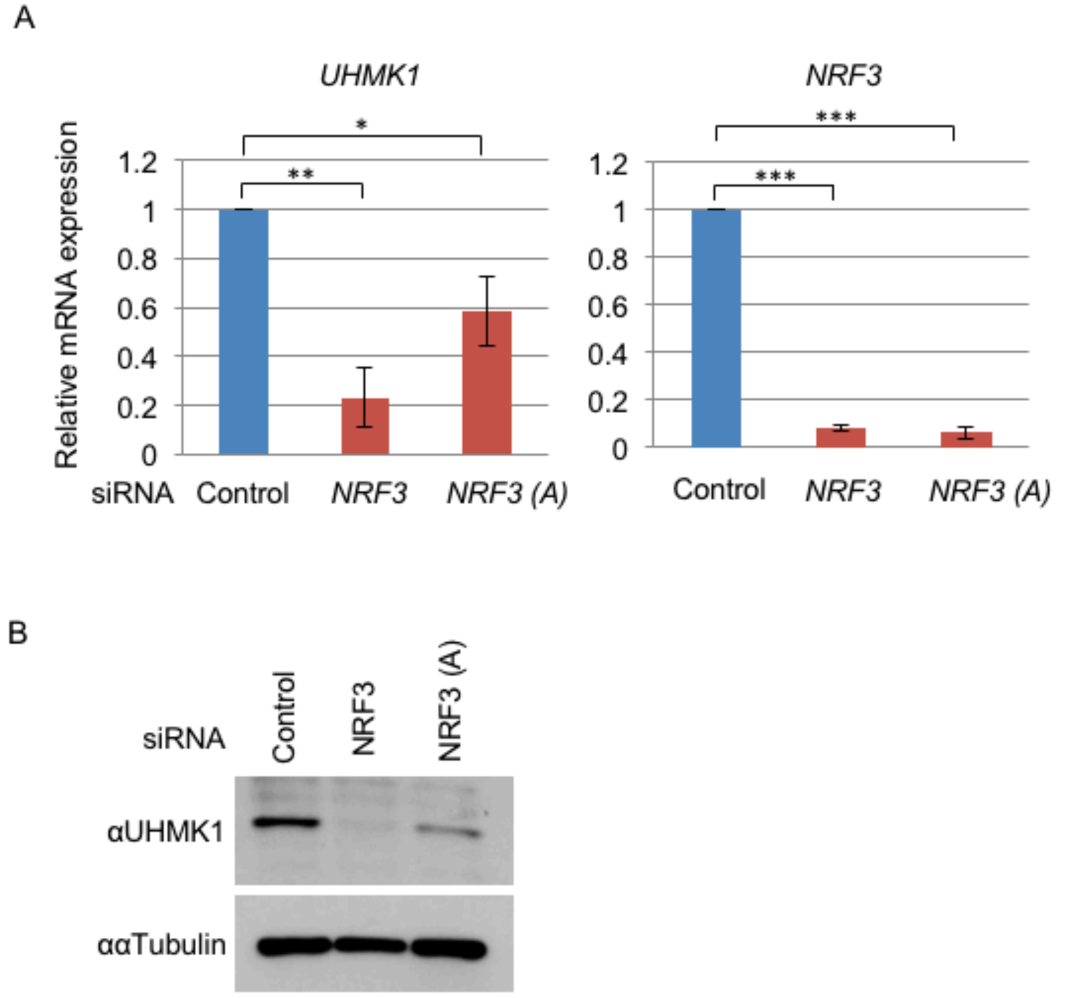

Figure S3. NRF3 also regulates the expression of *UHMK1* in HCT116 cell. (A and B) Reduction of the NRF3-mediated *UHMK1* expression was observed by introducing two distinct *NRF3* siRNAs into HCT116 cells. The mRNA and protein expression levels of UHMK1 were determined by qRT-PCR and immunoblot analyses, respectively. The detailed experimental procedure was described in the legend of Figures 5. The error bars (A) represent data from three independent experiments (mean ± standard deviation). The two-tailed Student's t-test was used for the statistical analysis. * $P < 0.05$, ** $P < 0.01$ and *** $P < 0.001$ compared to the Control data.

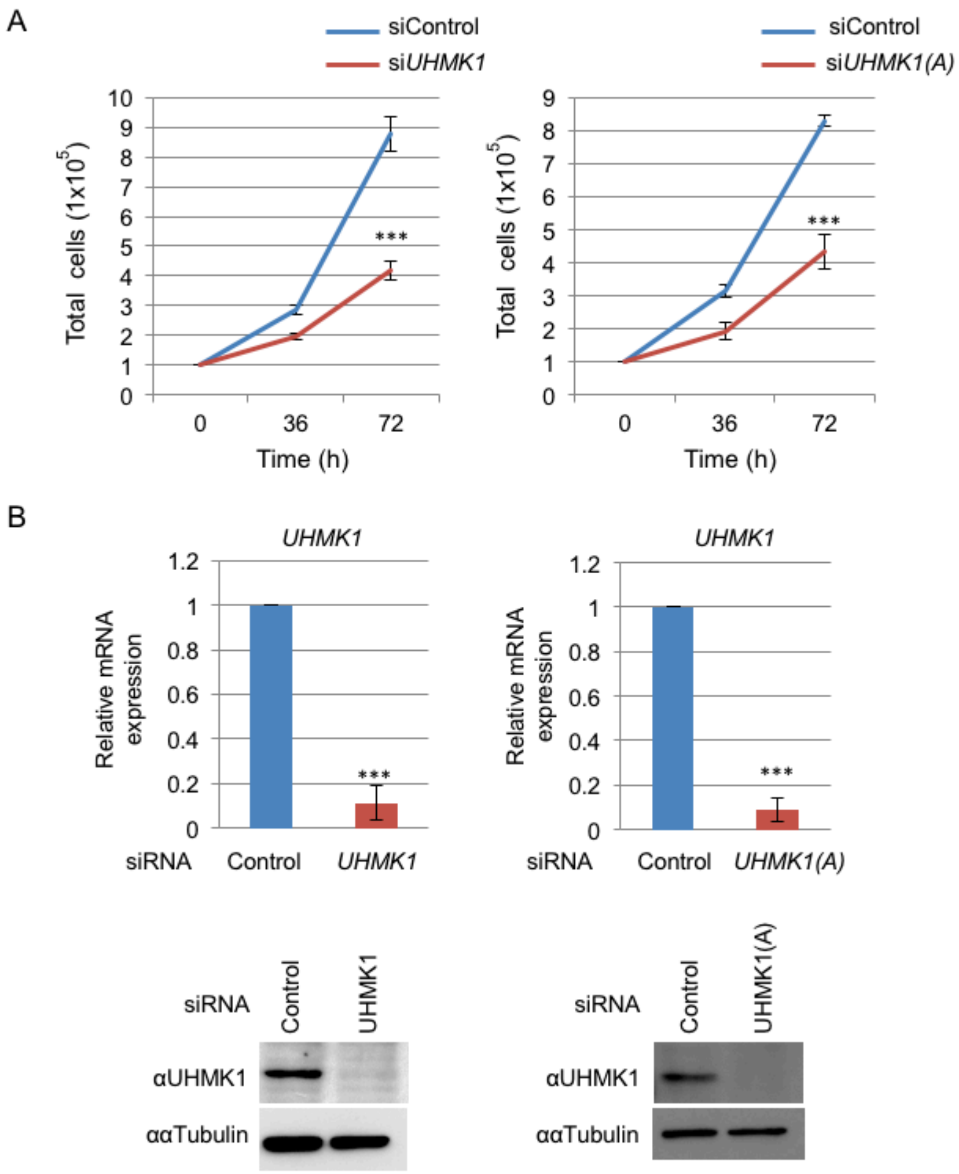

Figure S4. UHMK1 promotes the proliferation of colon cancer cells. (A) *UHMK1* knockdown significantly reduced the proliferation of DLD-1 cells. The cells were transfected with Control, *UHMK1 or UHMK1(A)* siRNA. At 36 and 72 hr after transfection, the cell numbers were counted using a hemocytometer. The initial cell numbers at the time of transfection were 1 x 10^5. (B) *UHMK1* siRNAs significantly reduces mRNA and protein levels of UHMK1 in DLD-1 cells. At 48 hr after transfection with Control, *UHMK1* or *UHMK1(A)* siRNA, the mRNA expression levels of *UHMK1* was determined by qRT-PCR analysis. The values were normalized to 18S rRNA data (top). Immunoblotting of the whole-cell extracts with the anti-UHMK1 antibodies was performed (bottom). α-Tubulin was used as an internal control. The error bars (A, B) represent data from two independent experiments in duplicate (n=4, mean ± standard deviation). The two-tailed Student's t-test was used for the statistical analysis. *** $P < 0.001$ (A, B) compared to the Control data.

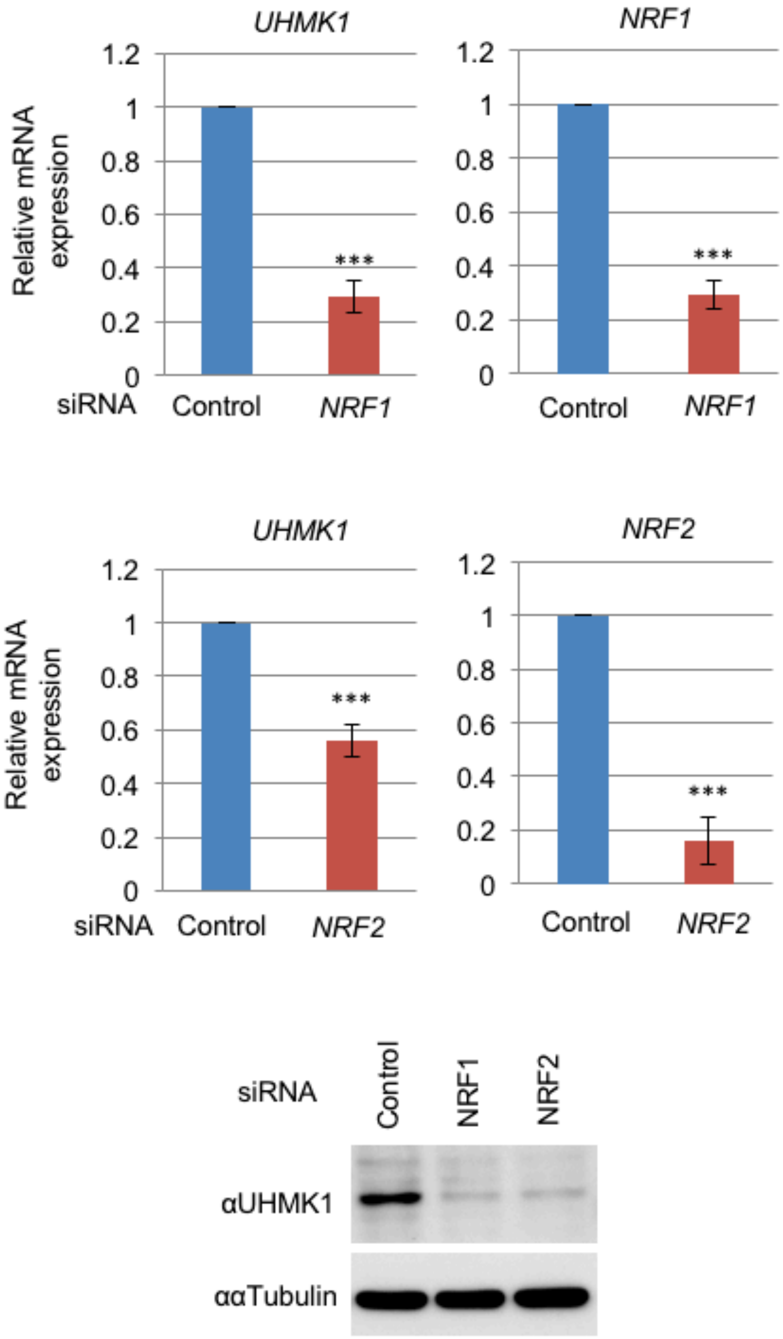

Figure S5. NRF1 and NRF2 also regulate the *UHMK1* gene expression. *NRF1 or NRF2* knockdown significantly reduce mRNA and protein levels of UHMK1 in DLD-1 cells. At 48 hr after transfection with Control, *NRF1* or *NRF2* siRNA, the mRNA expression levels of *UHMK1*, *NRF1 and NRF2* were determined by qRT-PCR analysis. The values were normalized to 18S rRNA data (top). Immunoblotting of the whole-cell extracts with the anti-UHMK1 antibodies was performed (bottom). α-Tubulin was used as an internal control. The error bars represent data from two independent experiments in duplicate (n=4, mean ± standard deviation). The two-tailed Student's t-test was used for the statistical analysis. *** $P < 0.001$ compared to the Control data.

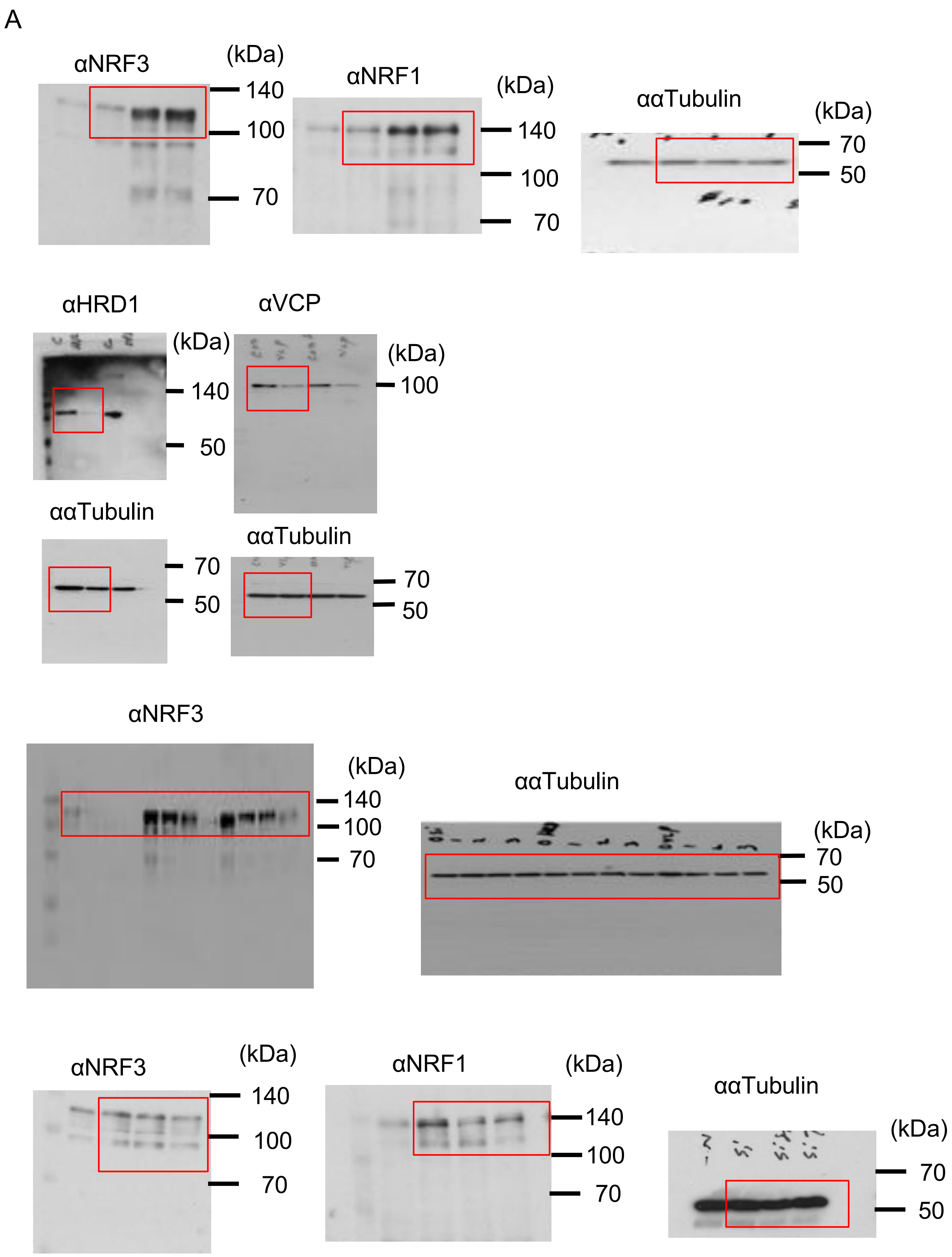

Figure S6 (A) Full-length western blots for figure 1 A, C, D and E. The line box regions are the cropped portion of the blot shown in the main figure.

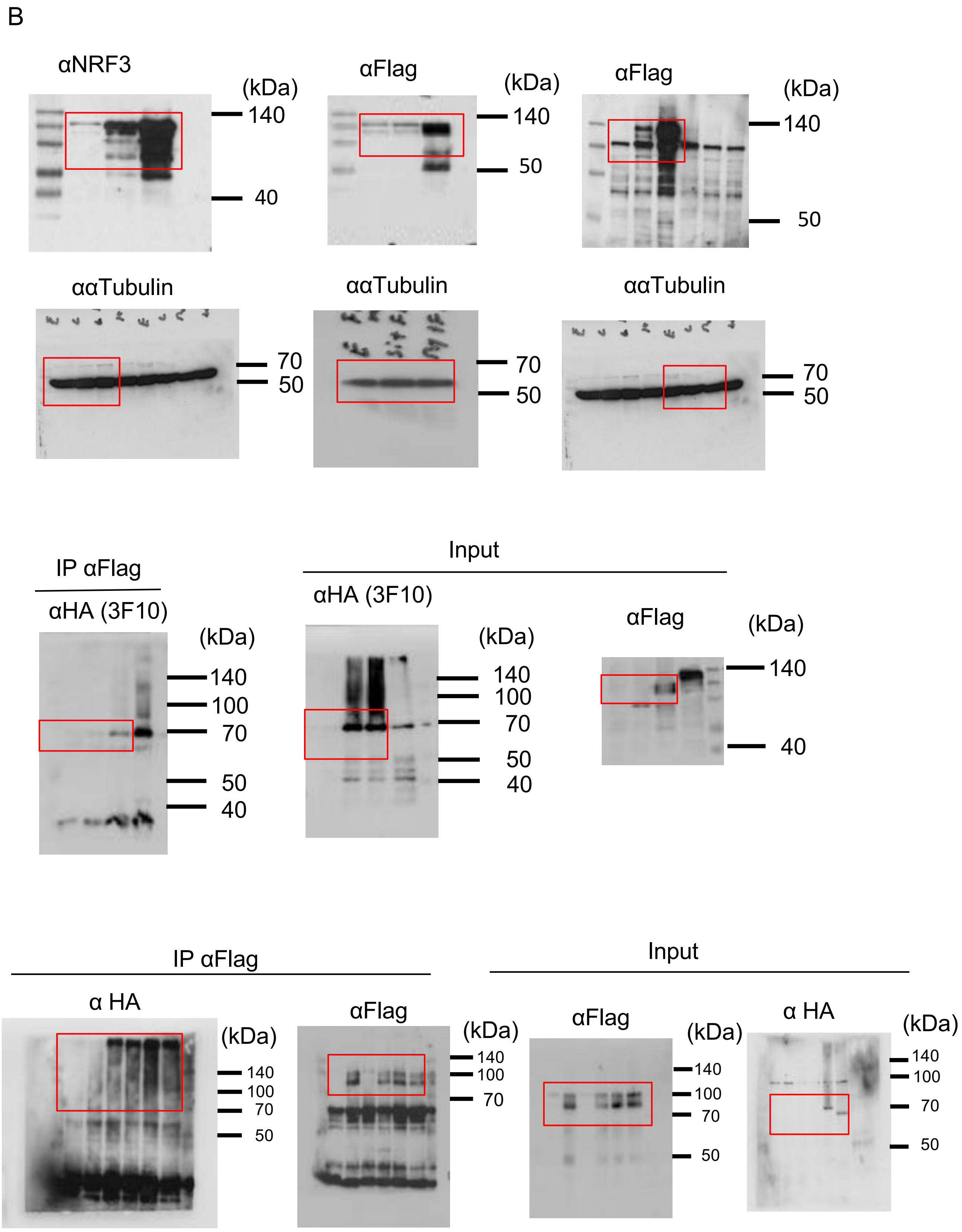

Figure S6 (B) Full-length western blots for figure 2A, C and D. The line box regions are the cropped portion of the blot shown in the main figure.

C

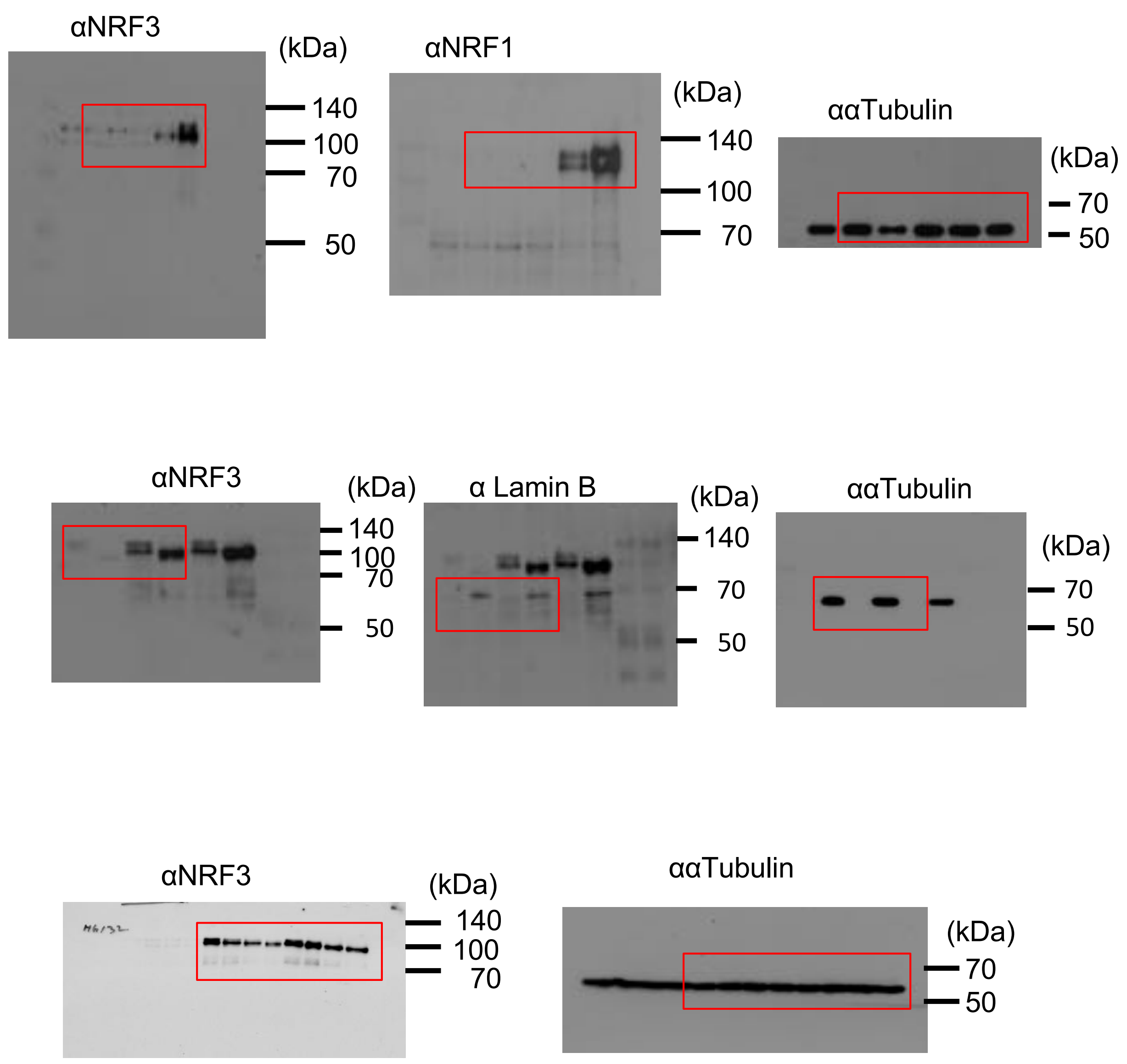

Figure S6 (C) Full-length western blots for figure 3A, C and E. The line box regions are the cropped portion of the blot shown in the main figure.

D

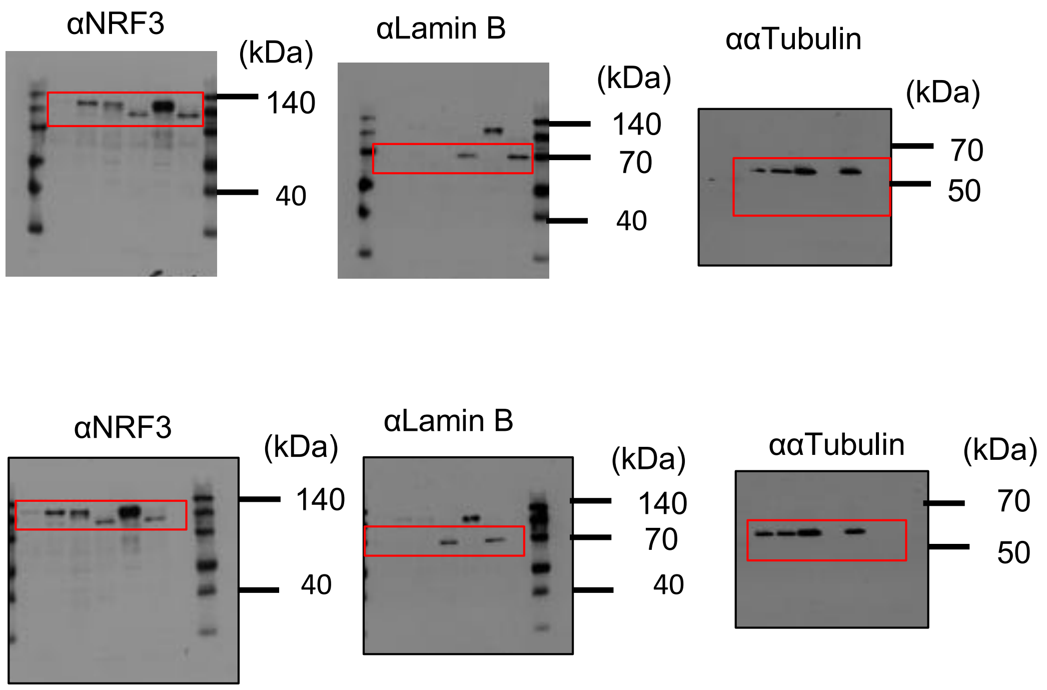

Figure S6 (D) Full-length western blots for figure 4 A and B. The line box regions are the cropped portion of the blot shown in the main figure.

E

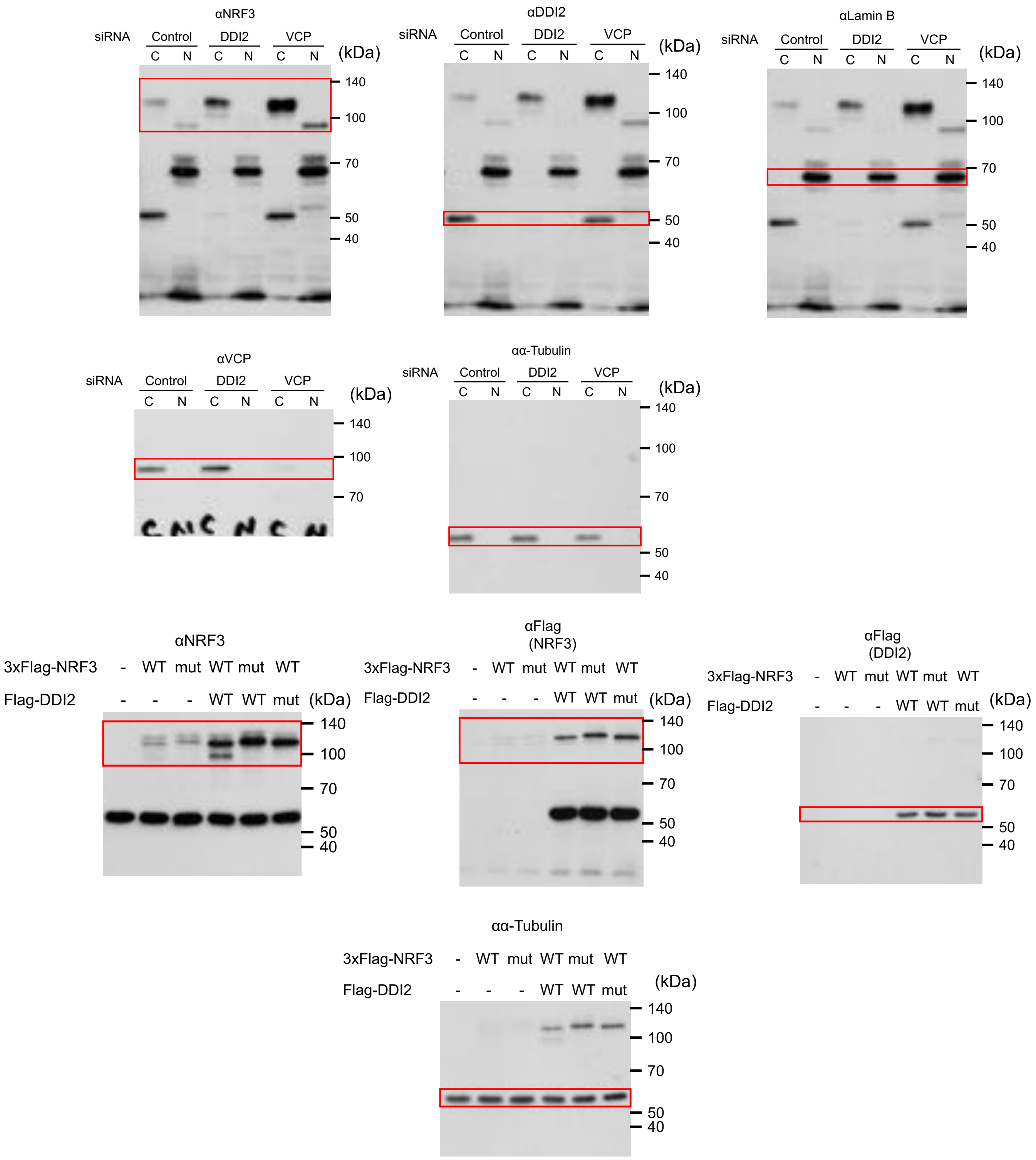

Figure S6 (E) Full-length western blots for figure 4 D and E. The line box regions are the cropped portion of the blot shown in the main figure.

F

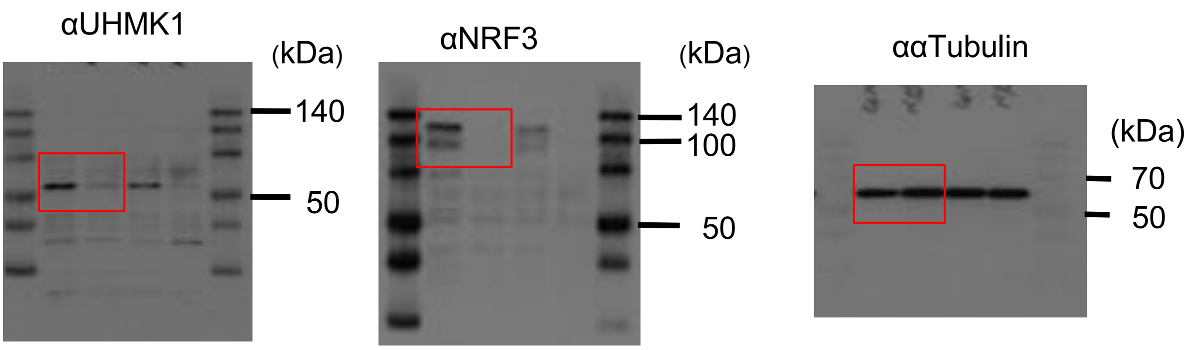

Figure S6 (F) Full-length western blots for figure 5C . The line box regions are the cropped portion of the blot shown in the main figure.

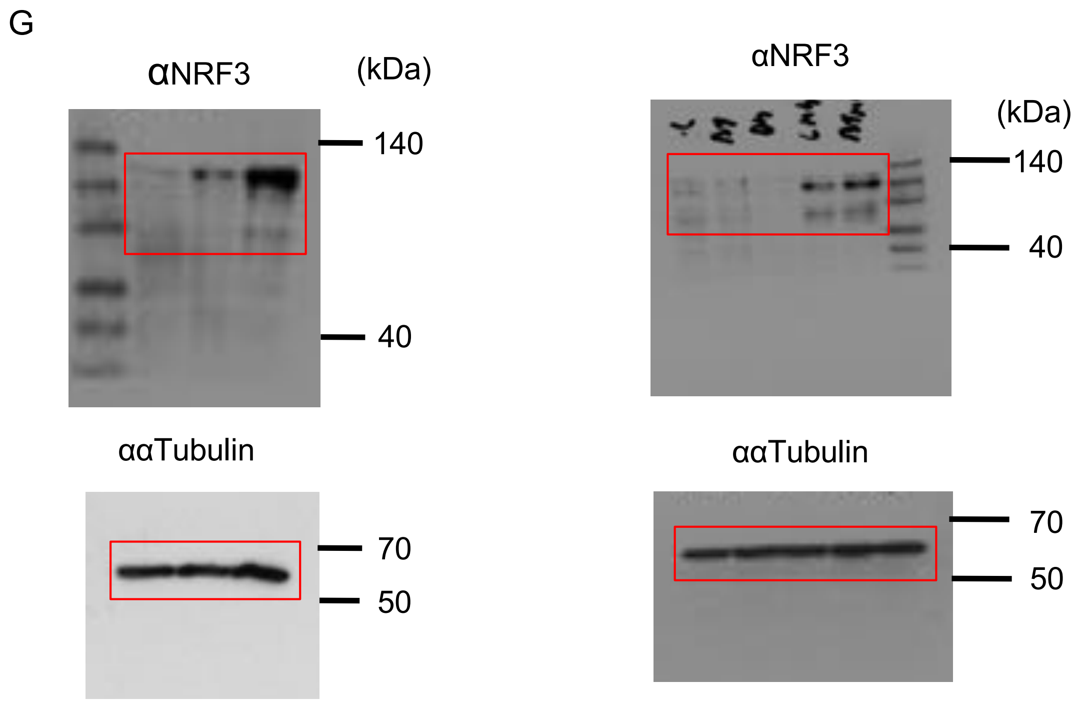

Figure S6 (G) Full-length western blots for figure S1 A and C. The line box regions are the cropped portion of the blot shown in the main figure.

H

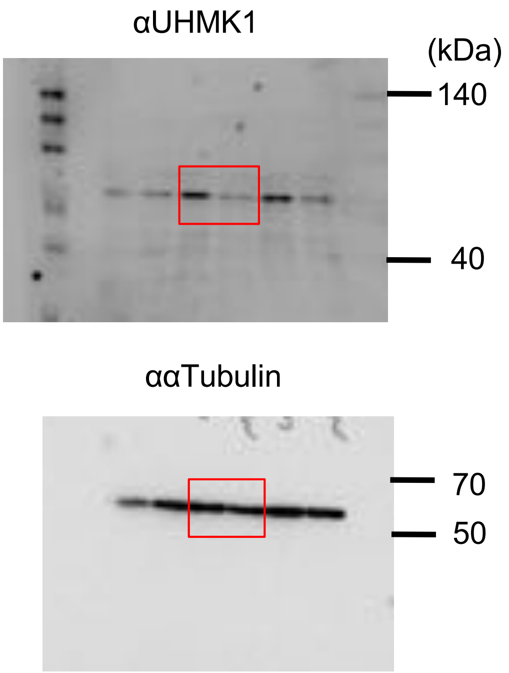

Figure S6 (H) Full-length western blots for figure S2 B. The line box regions are the cropped portion of the blot shown in the main figure.

I

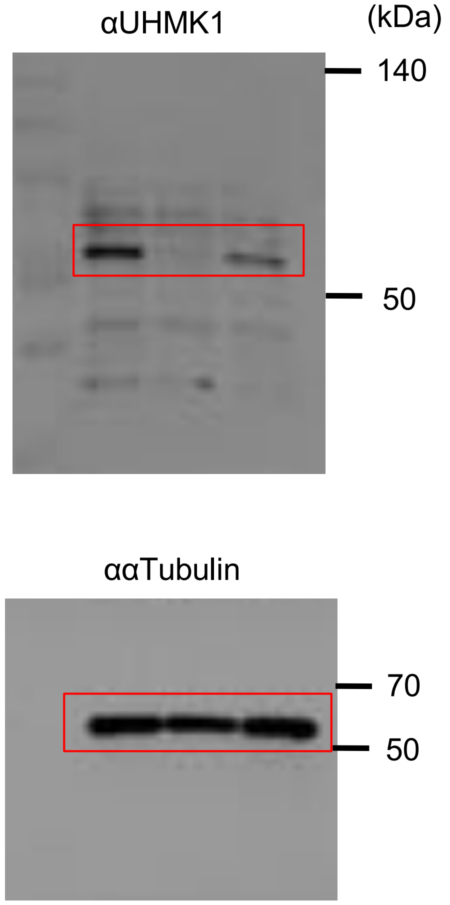

Figure S6 (I) Full-length western blots for figure S3 B. The line box regions are the cropped portion of the blot shown in the main figure.

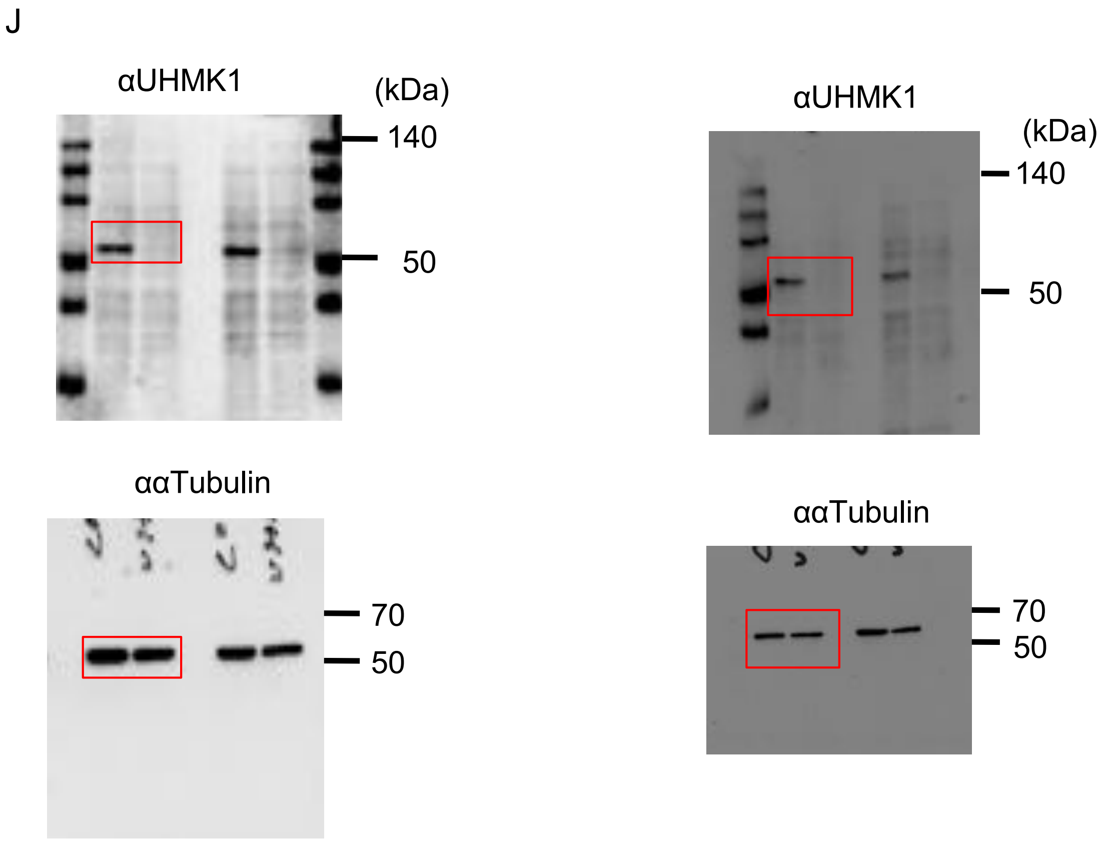

Figure S6 (J) Full-length western blots for figure S4 B. The line box regions are the cropped portion of the blot shown in the main figure.

K

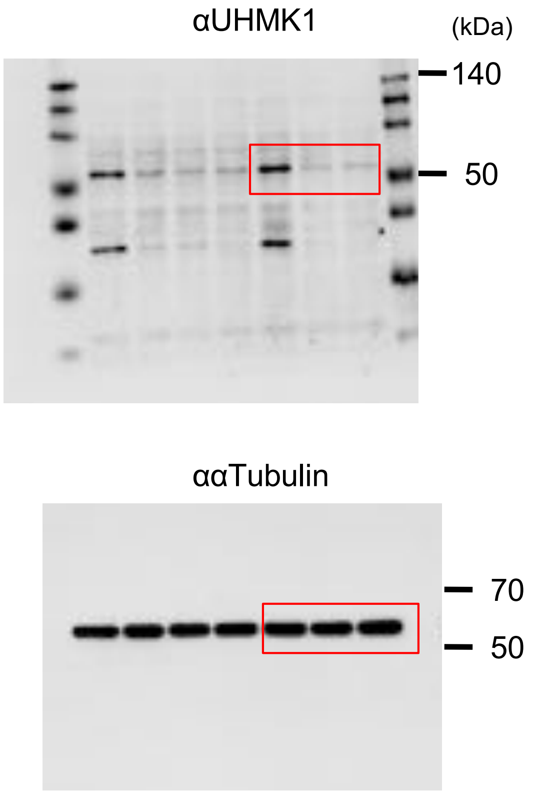

Figure S6 (K) Full-length western blots for figure S5. The line box regions are the cropped portion of the blot shown in the main figure.

Supplementary Table 1. Identification of NRF3-associated proteins by mass spectrometry.

Gene Symbol	Score	Protein Name/Description
SKP1	3	S-phase kinase-associated protein 1
VCP	4	transitional endoplasmic reticulum ATPase
USP15	4	ubiquitin carboxyl-terminal hydrolase 15
HCFC1	4	host cell factor 1
PSMA1	4	Proteasome subunit alpha type-1
PSMA2	4	Proteasome subunit alpha type-2
PSMA4	4	Proteasome subunit alpha type-4
PSMA6	4	Proteasome subunit alpha type-6
PSMA7	3	Proteasome subunit alpha type-7
PSMC1	4	26S Proteasome regulatory subunit 4
PSMC2	4	26S Proteasome regulatory subunit 7
PSMC3	4	26S Proteasome regulatory subunit 6A
PSMC6	4	26S Proteasome regulatory subunit 10B
PSMD11	4	26S Proteasome non-ATPase regulatory subunit 11
PSMD12	4	26S Proteasome non-ATPase regulatory subunit 12
PSMD2	3	26S Proteasome non-ATPase regulatory subunit 2
PSMD6	4	26S Proteasome non-ATPase regulatory subunit 6
PSMD8	4	26S Proteasome non-ATPase regulatory subunit 8
